# Transgenic rodents as dynamic models for the study of respiratory rhythm generation and modulation: a scoping review and a bibliometric analysis

**DOI:** 10.3389/fphys.2023.1295632

**Published:** 2023-12-21

**Authors:** Carol Alejandra Olmos-Pastoresa, Enrique Vázquez-Mendoza, María Leonor López-Meraz, César Antonio Pérez-Estudillo, Luis Beltran-Parrazal, Consuelo Morgado-Valle

**Affiliations:** Laboratorio de Neurofisiología, Instituto de Investigaciones Cerebrales, Universidad Veracruzana, Xalapa, Veracruz, Mexico

**Keywords:** respiratory rhythm, transgenic rodents, pre-Bötzinger complex, animal models, respiratory control

## Abstract

The pre-Bötzinger complex, situated in the ventrolateral medulla, serves as the central generator for the inspiratory phase of the respiratory rhythm. Evidence strongly supports its pivotal role in generating, and, in conjunction with the post-inspiratory complex and the lateral parafacial nucleus, in shaping the respiratory rhythm. While there remains an ongoing debate concerning the mechanisms underlying these nuclei’s ability to generate and modulate breathing, transgenic rodent models have significantly contributed to our understanding of these processes. However, there is a significant knowledge gap regarding the spectrum of transgenic rodent lines developed for studying respiratory rhythm, and the methodologies employed in these models. In this study, we conducted a scoping review to identify commonly used transgenic rodent lines and techniques for studying respiratory rhythm generation and modulation. Following PRISMA guidelines, we identified relevant papers in PubMed and EBSCO on 29 March 2023, and transgenic lines in Mouse Genome Informatics and the International Mouse Phenotyping Consortium. With strict inclusion and exclusion criteria, we identified 80 publications spanning 1997–2022 using 107 rodent lines. Our findings revealed 30 lines focusing on rhythm generation, 61 on modulation, and 16 on both. The primary *in vivo* method was whole-body plethysmography. The main *in vitro* method was hypoglossal/phrenic nerve recordings using the *en bloc* preparation. Additionally, we identified 119 transgenic lines with the potential for investigating the intricate mechanisms underlying respiratory rhythm. Through this review, we provide insights needed to design more effective experiments with transgenic animals to unravel the mechanisms governing respiratory rhythm. The identified transgenic rodent lines and methodological approaches compile current knowledge and guide future research towards filling knowledge gaps in respiratory rhythm generation and modulation.

## 1 Introduction

Breathing serves as a vital and evolutionary conserved behavior in vertebrates, particularly in mammals. This function, often referred to as eupnea, is essential for the gaseous exchange of oxygen (O_2_) and carbon dioxide (CO_2_) in the lungs, facilitated by the contraction of inspiratory muscles, such as the diaphragm (Li and Wang, 2021). Such an exchange is crucial for sustaining metabolic activities, regulating pH balance, and controlling body temperature (Del Negro et al., 2018). In mammals, the eupneic breathing cycle consists of three phases: Inspiration, post-inspiration, and expiration. Each phase arises from the synchronized activity of specialized neural circuits, known as central pattern generators (CPGs), located in the brainstem. Specifically, the pre-Bötzinger complex (preBötC) generated the inspiratory phase (Smith et al., 1991), the post-inspiratory complex (PiCo) is responsible for the post-inspiration phase (Anderson et al., 2016), and the lateral parafacial nucleus (pF_*L*_) controls the active expiration (Guyenet and Mulkey, 2010). These CPGs function in a mutually exclusive manner, coordinated by the preBötC, which acts as the central hub for respiratory rhythm (Del Negro et al., 2018).

The use of transgenic rodents has been pivotal in advancing scientific research since the 1970s for mice (Jaenisch and Mintz, 1974) and the 1990s for rats (Hammer et al., 1990; Mullins et al., 1990). Over 291,000 PubMed entries related to genetically modified animals, as of June 2023, underscore the extensive employment of these models. They offer a robust platform for studying the physiological mechanisms of breathing due to their genetic modifiability and the reproducibility of physiological phenomena using both *in vivo* and *in vitro* techniques.

Despite these advancements, a notable gap exists in the systematic identification of transgenic rodent lines and the compilation of methodologies used for studying respiratory rhythm. This absence is more than just an academic oversight; it represents a tangible limitation in the field. Without a comprehensive scoping review of the transgenic lines and methodologies employed, researchers face challenges in selecting the most appropriate tools for their studies. This lack of consolidated information can result in redundant efforts and may hinder the progress of research aimed at understanding respiratory rhythm in varying physiological states. It could also impact the translational potential of research findings, given that the choice of animal model and methodology can significantly influence the reproducibility and applicability of results.

Therefore, a scoping review was conducted to address these knowledge gaps. To execute this scoping review, the following research questions were formulated: What transgenic lines have been developed or utilized to investigate rhythm generation? Have these transgenic lines been used interchangeably to examine the generation and modulation of respiratory rhythm? What methodological approaches, including preparations and techniques, have been employed in these studies? The aim of this scoping review is to identify transgenic rodent lines commonly used for the study of respiratory rhythm generation and modulation and to compile the techniques and methodologies employed in this realm of research, including *in vivo* and *in vitro* techniques. By addressing this significant gap, we aim to contribute to developing a more comprehensive understanding of respiratory rhythm in both health and pathological states.

## 2 Material and methods

**FIGURE 1:**
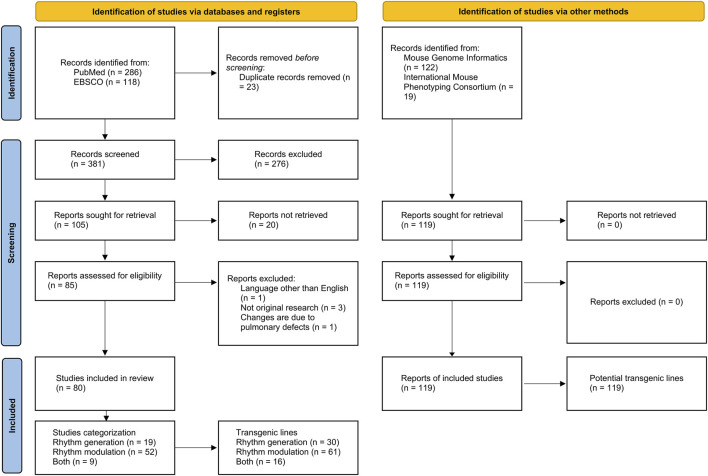
PRISMA flow diagram illustrating the scoping review process. Modified from Page et al. (2021).

This scoping review adhered to the PRISMA statement for scoping reviews (Tricco et al., 2018). We identified transgenic rodent lines used in respiratory generation studies using the PubMed and Scopus databases (Figure 1).

Furthermore, the Mouse Genome Informatics and the International Mouse Phenotyping Consortium databases helped us pinpoint potential transgenic mouse lines for respiratory rhythm generation and modulation investigations.

To address our research questions, we employed a modified version of the PIO (Population, Intervention, and Outcome) search strategy. Our search strategy centered on the utilization of “transgenic rodents” as the Population and examined its influence on the Outcome related to “respiratory rhythm.” The primary objective was to identify research papers that delve into the role of transgenic rodents in understanding the generation and modulation of respiratory rhythm. While the search did not specify a particular intervention or comparison, it concentrated on the intrinsic relationship between transgenic rodents and the outcome of interest, which is respiratory rhythm, within the available literature. To enhance the precision, comprehensiveness, and consistency of our search, we chose to incorporate MeSH (Medical Subject Headings) terms. MeSH terms are commonly employed in databases like PubMed to systematically index and categorize scientific literature. We inputted the following search terms into the PubMed and Scopus databases: (“Respiratory Mechanics” [MESH] OR “Respiratory Center” [MESH] OR “pre-Bötzinger”) AND (“Mice, Transgenic [MESH]” OR “Genetic Vectors” [MESH] OR “Animals, Genetically Modified” [MESH]). The distinction between the PubMed and EBSCO queries was the addition of *[MESH]* in PubMed to signify MeSH terms. In the transgenic mouse databases, we looked for alterations in respiratory rhythm using “abnormal pulmonary respiratory rate” and abnormal characteristics of the preBötC with “abnormal pre-Botzinger complex physiology” and “abnormal pre-Botzinger complex morphology,” which are annotated terms registered within the Mammalian Phenotype Ontology Annotation of the Mouse Genome Informatics database. This review included publications up to 29 March 2023, without year-based search filters.

We considered only original publications written in English. The inclusion criteria encompassed.

• Studies using transgenic rats or mice models showing changes in respiratory rhythm.
• Research employing viral vector injections (e.g., adeno-associated virus; AAV) into transgenic rodents to study respiratory rhythm or its changes.
• Studies implementing *in vivo* methods: plethysmography, tidal volume, and electromyography.
• Research using *in vitro* methods and preparations such as brainstem-spinal cord preparations/*en bloc*, working heart-brain stem preparation (WHB), rhythmic slice, whole-cell patch-clamp, intracellular or extracellular recordings, immunohistochemistry, histology, and electron microscopy.

The exclusion criteria consisted of.

• Studies on rodents other than rats and mice, like guinea pigs or hamsters.
• Research on mitochondrial respiration.
• Studies centered on mechanisms associated with lung or airway diseases.
• Systematic reviews, meta-analyses, or case reports.

Finally, the studies were categorized based on their utilization of animal models to investigate respiratory rhythm generation and/or modulation. The primary criterion for classification was whether the transgenic animals exhibited a failure to breathe (generation) or merely displayed alterations in breathing patterns (modulation).

**FIGURE 2:**
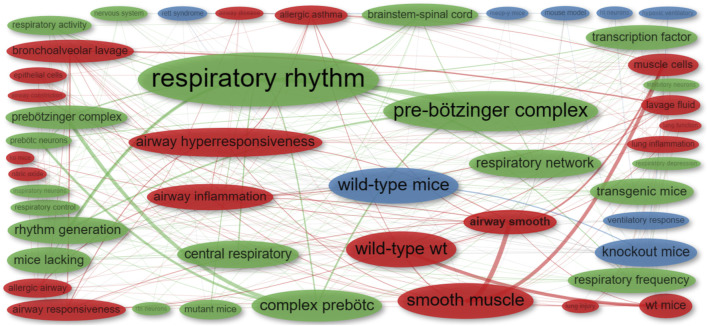
Co-occurrence network of bigrams extracted from the abstracts of publications identified in the initial phase. Distinct colors represent individual clusters. The frequency of each bigram is denoted by the size of its respective dot: larger dots signify more frequent bigrams. The co-occurrence strength between two bigrams is illustrated by the thickness of the connecting line: thicker lines indicate stronger co-occurrence.

To assess the relevance of our search for the scoping review, we conducted a preliminary bibliometric analysis, specifically examining bigram co-occurrences (co-word analysis) in abstracts from publications obtained from PubMed (Figure 2). This analysis entailed mapping and grouping bigram terms, which are two-word combinations (e.g., “pre-Bötzinger complex”), and identifying pairs of bigrams that frequently co-occur in a given context or dataset (e.g., “pre-Bötzinger complex” and “rhythm generation”). The first cluster (green) encompasses bigrams related to respiratory rhythm elements, the second (red) centers on elements of the respiratory apparatus, including central pattern generators (CPGs), and the third (blue) includes bigrams related to transgenic animals and disease models. This network indicates that most publications in the identification phase align with the objectives of this review.

### 2.1 Bibliometric analysis

We executed a bibliometric analysis on 80 retrieved publications using Bibliometrix (v.4.1.1) (Aria and Cuccurullo, 2017) in R (version 4.3.1—“Beagle Scout”) (R Core Team, 2022). We utilized KeyWords Plus and added synonyms. The co-occurrence network employed the “Star” layout with the Walktrap clustering algorithm and a repulsion force of 0.9. The Louvain clustering algorithm created the thematic map, and default parameters produced the remaining networks and plots. Thematic map themes are used as described in (Cobo et al., 2011).

## 3 Results

The literature search was conducted in PubMed and EBSCO databases, initially yielding 286 and 118 publications, respectively. After the elimination of 23 duplicate entries, a total of 381 publications were subjected to further scrutiny. Following the application of predefined inclusion and exclusion criteria, 276 publications were removed, leaving 105 publications for full-text retrieval. However, 25 of these were further excluded, resulting in a final selection of 80 publications for this review. These findings are visually summarized in Figure 1.

Transgenic lines were also identified through specialized repositories, specifically the Transgenic Mouse Databases and the International Mouse Phenotyping Consortium. This supplemental search resulted in 141 reports. After removing 22 duplicates, we proceeded to analyze 119 unique reports. These reports were subsequently categorized based on their focus on either the generation or modulation of respiratory rhythm, as depicted in Figure 1.

### 3.1 Transgenic rodent lines employed for studying respiratory rhythm generation and modulation: Techniques and approaches

Our scoping review of publications delving into respiratory rhythm generation and modulation, employing transgenic rodents as key subjects, has unveiled a multifaceted landscape of insights. These investigations have cast light on several pivotal dimensions, including the genes employed in the creation of transgenic lines, the age of animals utilized, the diverse *in vitro* and *in vivo* methodologies applied, electrophysiological preparations, the age of animals utilized for electrophysiological recordings, and the array of complementary techniques. Together, these findings form a comprehensive tapestry of knowledge, offering a deep and comprehensive understanding of the intricacies that govern respiratory rhythm in the context of experimental research.

#### 3.1.1 Gene identification in transgenic lines

Within the realm of respiratory rhythm research, the selection and manipulation of specific genes are pivotal in shaping our understanding of both rhythm generation and modulation. Some genes play a direct role in the generation of respiratory patterns, while others are utilized to precisely target and locate respiratory neurons. This section delves into the genes employed to generate transgenic lines, casting a spotlight on their dual significance in unraveling the intricacies of respiratory rhythm generation and modulation.

• **Rhythm Generation** In the context of rhythm generation, we conducted an analysis of 19 relevant publications. Our examination led us to the discovery of 17 unique genes utilized in the creation of 30 transgenic lines, as detailed in Table 1. These lines have proven instrumental in shedding light on the intricate mechanisms governing respiratory rhythm generation. Notably, the Dbx1 gene modification emerged as a prominent and frequently employed strategy, as evidenced by pivotal studies such as Wang et al. (2014) and Vann et al. (2016). An intriguing observation is that only a single study employed viral vectors and transgenic rats to manipulate gene expression within somatostatin-expressing neurons (Tan et al., 2008).
• **Rhythm Modulation** Our review encompassed 52 publications that engaged transgenic rodents in the exploration of rhythm modulation. In this realm, a total of 34 genes have been manipulated, resulting in the creation of 61 distinct transgenic lines, as summarized in Table 2. The *Mecp2* gene, specifically in the form of B6.129P2(C)-Mecp2^*tm*1.1*Bird*^, emerged as the most frequently targeted gene for modification. This gene was cited in multiple studies spanning from 2005 to 2021 (Viemari et al., 2005b; Bissonnette and Knopp, 2007; Stettner et al., 2007; Medrihan et al., 2008; Kron et al., 2011; Ward et al., 2011; Schmid et al., 2012; Dhingra et al., 2013; Matagne et al., 2021). Additionally, the utilization of various vectors, such as AAV-EF1*α*-DIO-hM3Dq-mCherry (Fu et al., 2019) and AAV9-MCO (Matagne et al., 2021), underscored the methodological diversity. It is worth noting that although the majority of publications employed transgenic mice, four publications conducted experiments on transgenic rats (Ikeda et al., 2015; Fu et al., 2019; Ikeda et al., 2019; Souza et al., 2020).
• **Rhythm Generation and Modulation** Our analysis extended to the study of both rhythm generation and modulation, examining nine relevant publications. Within this subset, we identified 11 genes that have been harnessed to generate 16 transgenic rodent lines, as detailed in Table 3. Interestingly, two publications employed viral vectors (Quintana et al., 2012; Vann et al., 2018), while one publication employed transgenic rats (Lladó et al., 2006).

**TABLE 1:** Genes used in generating transgenic rodent lines for studying respiratory rhythm generation.

Gene	Line	Type	Reference	Notes
Atp1a2	Atp1a2^−/−^	CKO	Ikeda et al. (2004)	Brain-specific due to gene expression pattern
Atoh1	Atho1^*Cre∕−*^; Tau^*mgfp−nLacZ*^	CKO	Huang et al. (2012)	Expression in hindbrain during development
	Atho1^*Phox*2*bCKO*^	CKO		Expression preferentially in retrotrapezoid nucleus
	Atoh1^*HoxA4CKO*^	CKO		Expression in caudally derived Atho1 lineages
chx10	Chx10:LNL:DTA	CKO	Crone et al. (2012)	Ablation of V2a neurons by diphtheria toxin A-chain (DTA)
Dbx1	Dbx1^+/*CreERT*2^; Rosa26^*tdTomato*^	CKI	Wang et al. (2014)	Identification of Dbx1 neurons
GluR3	GluR3^−/−^	KO	Steenland et al. (2008)	Constitutive knockout
Jmjd3	Jmjd3^−/−^	KO	Burgold et al. (2012)	Neurogenic factor required for maintenance of the preBötC
	Jmjd3^−/−^; Jmjd3^*mut*^21	KO		Point mutation A1388H
	Jmjd3^−/−^; Jmjd3^*mut*^65	KO		Point mutation A1388H
	Jmjd3^−/−^; Rosa26^*Jmjd*3^	KO/CKI		Conditional rescue of Jmjd3
	Rosa26^*flSTOP*−*Jmjd*3^	CKI		Conditional expression of Jmjd3
Lbx1	Lbx1^*GFP∕GFP*^	KO	Pagliardini et al. (2008)	Expression during development of medulla and spinal cord
MAO-A	Tg8	KO	Bou-Flores et al. (2000)	Deletion of gene. Displays excess of 5-HT.
Mafb	Mafb^−/−^	KO	Blanchi et al. (2003)	Constitutive knockout. Differentiation of hematopoietic system, podocytes, and specification of rhombomeres in the early hindbrain
Mecp2	B6.129P2(C)-MeCP2^*tm*1−1*Bird*^	KO	Mironov et al. (2009) ^■^	Constitutive knockout. Transduction of neurons in organotypic slices with a Ca^2+^sensor (D3cpv) via AAV.
Rnx	Rnx^−/−^	KO	Shirasawa et al. (2000)	Orphan homeobox gene expressed in the developing dorsal and ventral region of the medulla
Sst	Sprague-Dawley	KI	Tan et al. (2008) ^★^^■^	Transduction with AAV2-pA-Sst-alstR-IRES-EGFP or AAV2-pA-Sst-EGFP into the preBötC is performed to express the allatostatin receptor and EGFP (enhanced green fluorescent protein) in Sst neurons. Alternatively, only EGFP is expressed in Sst neurons using AAV2-pA-Sst-EGFP.
STIM1	STIM1^−/−^	KO	Berry et al. (2003)	Highly expressed in the central nervous system and placenta
Thy1.2	Thy1.2-EYFP	KI	Winter et al. (2007)	Expression of EYFP (enhanced yellow fluorescent protein) in the ventral respiratory column, preBötC, and the rostral ventral respiratory group neurons
	Thy1.2-HcRed	KI		Expression of HcRed in the ventral respiratory column, preBötC, and the rostral ventral respiratory group neurons
Tshz3	Tshz3^+/*lacZ*^	KO	Caubit et al. (2010)	Transcription factor expressed in the brainstem
Vglut2	Vglut2^*f*/*f*;*PCre*^	CKO	Wallén-Mackenzie et al. (2006)	First model showing complete uncompensated functional impairment of the respiratory CPG.
Vglut2, Dbx1, and Sst	Dbx1-tdTomato	CKI	Koizumi et al. (2016)	Expression of tdTomato in Dbx1-neurons
	Dbx1-tdTomato-Arch-GFP	CKI		Expression of tdTomato, archaerhodopsin (Arch), and GFP in Dbx1-neurons
	Sst-tdTomato	CKI		Expression of tdTomato in Sst-expressing neurons
	SST-tdTomato-Arch-GFP	CKI		Expression of tdTomato, Arch, and GFP in Sst-expressing neurons
	VgluT2-tdTomato	CKI		Expression of tdTomato in VgluT2-expressing neurons
	VgluT2-tdTomato-Arch-GFP	CKI		Expression of tdTomato, Arch, and GFP in VgluT2-expressing neurons
	VgluT2-tdTomato-Gad67-GFP	CKI		Expression of tdTomato in VgluT2-expressing neurons and expression of GFP in Gad67-expressing neurons
ZFHX	Zfhx4^*PB*/+^	KO	Zhang et al. (2021)	Transcriptional factor expressed in the brainstem

Unless stated, all transgenic lines are from mice. ^★^Transgenic rats. ^■^Viral vectors. Atoh1/Math1 (Atonal BHLH, Transcription Factor 1), Atp1a2 (ATPase, Na+/K + Transporting Subunit Alpha 2), Chx10 (Visual system homeobox 2), Dbx1 (Developing Brain Homeobox 1), Gad67 (Glutamate Decarboxylase 67 KDa, Isoform), GluR3 (Glutamate Ionotropic Receptor AMPA, Type Subunit 3), Jmjd3 (Jumonji domain-containing protein-3), Lbx1 (Ladybird Homeobox 1), Mafb (MAF BZIP, Transcription Factor B), Maoa (Monoamine oxidase A), Mecp2 (Methyl-CpG, Binding Protein 2), Rnx (Respiratory Neuron Homeobox), Sst (Somatostatin), Stim1 (Stromal Interaction Molecule 1), Thy1 (Thy-1, cell surface antigen), Tshz3 (Teashirt Zinc Finger Homeobox 3), Vglut2 (Vesicular glutamate transporter 2), and Zfhx3 (Zinc Finger Homeobox 3).

KO, knockout; KI, knock-in; CKO, conditional knockout; CKI, conditional knock-in.

**TABLE 2:** Genes used in generating transgenic rodent lines for studying respiratory rhythm generation and modulation.

Gene	Line	Type	Reference	Notes
A*β*PP	FVB-Tg	KI	Andrzejewski et al. (2022)	Constitutive transgenic overexpressing human A*β*PP with V717I mutation under the control of a fragment of thy1 promoter
*α*SYN	PLP-*α*SYN	KI	Flabeau et al. (2014)	Expression of wild-type *α*-synuclein in oligodendrocytes under the control of the proteolipid promoter (PLP)
Chrna4	L9′A	KI	Shao et al. (2008)	Constitutive knock-in with a point mutation at the Leu9′ position in the M2 pore-lining region of nAChR *α*4
DMPK	DM600	KI	Panaite et al. (2013)	Constitutive knock-in carrying 600 CTG repeats in human DMPK.
	DMSXL	KI		Constitutive knock-in carrying more than 1300 CTG repeats in human DMPK.
Dbx1	Dbx1^*CreERT*2^; Rosa26^*tdTomato*^	CKI	Kottick and Del Negro (2015)	Expression of tdTomato in Dbx1 neurons
	Dbx1^*CreERT*2^; Rosa26^*hChR*2(*H*134*R*)−*tdTomato*^	CKI		Expression of channelrhodopsin in Dbx1 neurons
Dbx1 and SST	Dbx1-Cre; ChR2(H134R)-tdTomato	CKI	Cui et al. (2016) ^■^	Expression of channelrhodopsin in Dbx1 neurons
	Sst-IRES-Cre; ChR2(H13R)-tdTomato	CKI		Expression of channelrhodopsin in Sst neurons
	Sst-cre	CKI		Mice injected with AAV2/1-Ef1*α*-DIO-ChR2-eYFP into preBöt
Epo	Epo-Tag^*h*^	KO	Macarlupú et al. (2006)	Mice exhibiting a reduced EPO expression. Animal model of chronic anemia
Gaa	Gaa^−/−^	KO	DeRuisseau et al. (2009)	Constitutive knockout
	hGAA (MTP)	KO		Muscle-specific knockout
GDNF	GDNF^−/−^	KO	Huang et al. (2005)	Constitutive knockout
GLS1	GLS^−/−^	KO	Masson et al. (2006)	Constitutive knockout
H1R	H1RKO	KO	Miyamoto et al. (2004)	Constitutive knockout
KCC2a	KCC2a^−/−^	KO	Dubois et al. (2018)	Brain-specific due to gene pattern expression
Kir4.1	Kir4.1^−/−^	KO	Neusch et al. (2006)	Astrocyte-specific through the inbreeding of Kir4.1^−/−^and TgN (hFGAP-EGFP)
Lmx1b	Lmx1b^*f*/*f*/*p*^	CKO	Corcoran et al. (2014); Hodges et al. (2009)	Selectively lack serotonin neurons (Lmx1b was only deleted in Pet1-expressing 5-HT neurons)
Mafb	kr/kr	KO	Chatonnet et al. (2002)	Generated through X-ray-induced mutation, it affects only the early hindbrain expression of Mafb
MAOA	Tg8 [Tg (H2-IFN-*β*)8]	KO	Bou-Flores and Hilaire (2000); Viemari and Hilaire (2003)	Constitutive knockout
Math1	Math1^Cre^*^PR^	CKO	Rose et al. (2009)	Constitutive conditional knockout
	Math1^Cre^*^PR/+^; Rosa^*EYFP∕+*^; Tau^*mGFP∕+*^	CKI		This allows for the visualization of cell somas (EYFP) and their projections (mGFP) in Math1-derived populations in the medulla
	Math1^*M1GFP∕M1GFP*^	KI		Math1 tagged with EGFP.
	Math1^*LacZ∕LacZ*^	KO		Constitutive knockout
Mecp2	Mecp2^*tm*1.1*Jae*^	KO	Schmid et al. (2012); Dhingra et al. (2013)	Constitutive knockout. They do not express the full MeCP2 protein, but they do express smaller peptides
	B6.129P2(C)-Mecp2^*tm*1.1*Bird*^	KO	Kron et al. (2011); Medrihan et al. (2008); Bissonnette and Knopp (2007); Viemari et al. (2005b); Ward et al. (2011); Voituron et al. (2010); Stettner et al. (2007); Matagne et al. (2021) ^■^	Constitutive knockout. There is a complete loss of both the gene and its corresponding protein. Matagne et al. (2021)generated a gene therapy using an AAV-MCO vector to restore MeCP2
	Mecp2^*flox∕+*^; HoxB1^*cre*^	CKO	Ward et al. (2011)	Regional knockout of MeCP2 within the HoxB1 region
	Mecp2^*Tm*2*Bird*^; HoxB1^*cre*^	CKI		Restoring of MeCP2 within the HoxB1 domain
	Mecp2^*Tm*1*Jae*^; HoxB1^*cre*^	CKO		Regional knockout of MeCP2 within the HoxB1 region
Mrgpra1	B6.Cg-Tg (hGFAP-tTA:tetO-MrgA1)1^1*Kdmc*/*Mmmh*^	KI	Forsberg et al. (2017)	Expression of GFP and MrgA1 receptor (MrgA1) in astrocytes (GFAP) in the brainstem. MrgA1 receptor is normally not expressed in the brain
NMDAR1	NMDAR1^−/−^	KO	Funk et al. (1997)	Constitutive knockout
Nurr1	Nurr1^−/−^	KO	Nsegbe et al. (2004)	Constitutive knockout
ORX/ATX	ORX/ATX-Tg	KO	Toyama et al. (2009)	Constitutive knockout
PACAP	PACAP^−/−^	KO	Arata et al. (2013)	Constitutive knockout. Brain-specific due to gene pattern expression
Pet-1	Pet-1^−/−^	KO	Hodges et al. (2011)	Constitutive knockout. It lacks 70% of 5-HT neurons
Phox2b	Phox2b-EYFP/CreER^*T*2^	CKI	Ikeda et al. (2015) ^★^	Generated to express EYFP and Cre-recombinase estrogen receptor T2 in Phox2b neurons
	Phox2b-Cre	CKI	Fu et al. (2019) ^■^	Transduction with AAV-EF1*α*-DIO-hM3Dq-mCherry or AAV2-EF1*α*-DIO-mCherry into the nucleus tractus solitarii. To express mCherry and human M3 muscarin receptor (hM3Dq)
	Phox2b-Arch	KI	Ikeda et al. (2019) ^★^	Expression of archaerhodopsin-3 in Phox2b-expressing cells
	Phox2b^27*Ala*/+^	CKO	Dubreuil et al. (2009)	Most frequent human PHOX2B mutation
	Phox2b^*lox∕lox*^; Egr2^*cre∕+*^	CKO		Regional knockout within the Egr2 region
	Phox2b^*lox∕lox*^; Islet1^*cre∕+*^	CKO		Regional knockout within the Islet1 region (motoneurons precursors)
	Phox2b^*lox∕lox*^; Lbx1^*cre∕+*^	CKO		Regional knockout within the Lbx1 region
Phox2b and Atoh1	Phox2b^Δ8^; Atoh-1^*cre*^	CKI	Ferreira et al. (2022)	Conditional mutation of Phox2b in Atoh1-expressing cells
Ret	Ret^−/−^	KO	Viemari et al. (2005a)	Hindbrain-specific due to gene pattern expression
Slc6a5	Tg (Slc6a5-icre)121Veul; 129-Gt (ROSA) 26Sor^*tm*32(*CAG*−*COP*4^*^H134*R*/*EYFP*)*Hze*/*J*^	CKI	Hülsmann et al. (2021)	Expression of channelrhodopsin in inhibitory neurons (glycine transporter 2 promoter)
	Tg (Slc6a5-icre)^121*Veul*^	CKI	Fortuna et al. (2019) ^■^	Transduction with pAAV-EF1*α*-DIO-hChR2(H134R)-EYFP-WPRE-HGHpA into the BötC and the rostral preBötC to express channelrhodopsin and EYFP in glycinergic neurons
Tac1	Tac1^−/−^	KO	Berner et al. (2007)	Constitutive knockout
Tacr1	${NK}_{1}^{-/-}$	KO	Ptak et al. (2000)	Constitutive knockout
Tau	Tau.P301L	KI	Menuet et al. (2011)	Neuron-specific due to thy1 gene promoter. Expression of the longest human tau isoform with the P301L mutation
TH	TH-cre	CKO	Souza et al. (2020) ^★^■	Transduction with LVV-PRSx8-ChR2(H134R)-mCherry and rAAV5-Flex-taCaspase2-Tevp into the retrotrapezoid nucleus is performed to express channelrhodopsin and mCherry and to ablate C1 neurons. Alternatively, transduction with rAAV2-eF1*α*-DIO-hChR2 is used to selectively express channelrhodopsin in C1 neurons
UBB	C57BL/6-Tg (Camk2a-UBB)3413-1Fwvl/J	KI	Irmler et al. (2012)	Overexpression of mutant ubiquitin B, driven by the CamKII*α*promoter (excitatory neuron-specific)
Vgat	L7^*cre*^;Vgat^*flox*7*flox*^	CKO	Liu et al. (2020)	Selective loss of Purkinje cell (L7/Pcp2 promoter) GABAergic synaptic transmission
	Vgat-tdTomato	KI	Ausborn et al. (2018)	Expression of tdTomato in Vgat neurons
	Vgat-tdTomato-ChR2-EYFP	KI	Ausborn et al. (2018)	Expression of tdTomato and channelrhodopsin in Vgat neurons
Vglut2, Dbx1, Sst, and Vgat	Vglut2cre^*Ai*32^	CKI	Huff et al. (2022)	Expression of channelrhodopsin fused to an EYFP (Ai32) in glutamatergic neurons
	Dbx1cre^*Ai*32^	CKI		Expression of channelrhodopsin fused to an EYFP (Ai32) in Dbx1 neurons
	Dbx1cre^*Ai*40*D*^	CKI		Expression of archaerhodopsin fused to an EGFP (Ai40D) in Dbx1 neurons
	Sstcre^*Ai*32^	CKI		Expression of channelrhodopsin fused to an EYFP (Ai32) in Sst neurons
	Vgatcre^*Ai*32^	CKI		Expression of channelrhodopsin fused to an EYFP (Ai32) in inhibitory neurons

Unless stated, all transgenic lines are from mice. ^★^Transgenic rats. ^■^Viral vectors. A*β*PP (Amyloid-*β*protein precursor), Atho1/Math1 (Atonal BHLH, Transcription Factor 1), *α*SYN (Alpha-synuclein), Chrna4 (Cholinergic Receptor Nicotinic Alpha 4 Subunit), Dbx1 (Developing Brain Homeobox 1), Dmpk (Dystrophia myotonica protein kinase), Epo (Erythropoietin), Gaa (Acid alpha-glucosidase), Gdnf (Glial Cell Derived Neurotrophic Factor), Gls1 (Glutaminase 1), Hrh1 (Histamine Receptor H1), Kcc2a (Neuronal isoform of the K-Cl cotransporter 2), Kir4.1/KCNJ10 (Potassium inwardly rectifying channel subfamily J member 10), Lmx1b (LIM, homeobox transcription factor 1-beta), MafB (MAF BZIP, Transcription Factor B), Maoa (Monoamine oxidase A), Mecp2 (Methyl-CpG, Binding Protein 2), Mrgpra1 (Mas-related G-protein coupled receptor member A1), Nmdr1 (N-methyl-D-aspartate receptor subunit NR1), Nurr1 (Nuclear receptor 4A2), ORX/ATX (Orexin/ataxin-3), Pacap (Pituitary adenylate cyclase-activating polypeptide), Pet-1 (pheochromocytoma 12 ETS (E26) transformation-specific factor), Phox2b (Paired Like Homeobox 2B), Ret (Ret Proto-Oncogene), Slc4a3 (Solute Carrier Family 4 Member 3), Slc6a5 (Solute Carrier Family 6 Member 5), Scnn1b (Sodium Channel Epithelial 1 Subunit Beta), Sst (Somatostatin), Tac1 (Tachykinin Precursor 1), Tacr1 (Tachykinin Receptor 1), Tau/Mapt (Microtubule Associated Protein Tau), Th (tyrosine Hydroxylase), Ubb (Ubiquitin B), and Vgat (Vesicular GABA, transporter).

KO, knockout; KI, knock-in; CKO, conditional knockout; CKI, conditional knock-in.

**TABLE 3:** Genes used in generating transgenic rodent lines for studying respiratory rhythm generation and modulation.

Gene	Type	Line	Reference	Notes
Atp1a2	Atp1a2^−/−^	KO	Onimaru et al. (2007)	Constitutive knockout. Gene expresses specifically and abundantly in skeletal muscle, heart, and brain
bdnf and nt-4	bdnf^−/−^	KO	Balkowiec and Katz (1998)	Constitutive knockout
	nt-4^−/−^	KO		Consitutive knockout
Dbx1	Dbx1; ArchT	KI	Vann et al. (2018) ^■^	Expression of archaerhodopsin fused to EGFP in Dbx1 neurons
	Dbx1; CatCh	KI		Expression of calcium translocating channelrhodopsin and EYFP in Dbx1 neurons. Transduction of AAV-eSyn-FLPo into the preBötC to specifically limit the expression of CatCh to the preBötC area
Hoxa2 and Krox20	Hoxa2^−/−^	KO	Chatonnet et al. (2007)	Constitutive knockout
	Hoxa2^*EGFP(lox−neo−lox)*^; r2; Cre	CKI		Expression of EGFP in r2-derived Hoxa2 expressing cells
	Krox20^−/−^	KO		Constitutive knockout
	Krox20^*Cre∕flox*^	CKI		Transient expression of Krox20 in the hindbrain
Dscam	Dscam^−/−^	KO	Amano et al. (2009)	Constitutive knockout. Brain-specific due to gene pattern expression
Mecp2	B6.129P2(C)-Mecp2^*tm*1.1*Bird*^	KO	Voituron et al. (2010)	Constitutive knockout
Ndufs4	NesKO	CKO	Quintana et al. 2012) ^■^	Neuron-specific (nestin) knockout
	Ndufs4-KO	KO		Constitutive knockout. Some animals received gene therapy delivered by an AAV1-Ndufs-IRES-GFP vector
	Ndufs4^*lox∕lox*^	CKO		Transduction with AVV1-Cre-GFP to selectively knockout of Ndusf4 in the vestibular nucleus
SOD1	SOD1^*G*93*A*^	KI	Lladó et al. (2006) ^★^	Expression of a mutant SOD1
Spi1	Spi1^−/−^	KO	Cabirol et al. 2022)	Model lacking microglia. They also used a Cx3xr1^*GFP*^, in which microglia express GFP.

Unless stated, all transgenic lines are from mice. ^★^Transgenic rats. ^■^Viral vectors. Atp1a2 (ATPase, Na+/K + Transporting Subunit Alpha 2), Bdnf (Brain-Derived Neurotrophic Factor), Dbx1 (Developing Brain Homeobox 1), Dscam (DS, Cell Adhesion Molecule), Hoxa2 (Homeobox A2), Krox20 (Early growth response protein 2), Mecp2 (Methyl-CpG, Binding Protein 2), Ndufs4 (NADH, Ubiquinone Oxidoreductase Subunit S4), Nt-4 (Neurotrophin 4), Sod1 (Superoxide Dismutase 1), Spi1 (Spi-1, Proto-Oncogene).

**Evaluation:** These studies have elucidated crucial aspects of both rhythm generation and modulation, shedding light on the genetic and mechanistic underpinnings of respiratory control. Notably, the prominent role of the Dbx1 gene in rhythm generation and the prevalence of the *Mecp2* gene in modulation underscore the significance of these genes in respiratory research. Additionally, the limited use of transgenic rats, despite their potential, suggests a promising avenue for further exploration and research diversification in this field.

#### 3.1.2 *In vitro* and *in vivo* approaches and techniques

Our examination of the literature has unveiled a mosaic of methodological approaches employed across publications in these domains. This exploration reveals the multifaceted nature of research in respiratory rhythm. The results presented here offer valuable insights into the diversity of methodological choices made by researchers in the exploration of respiratory rhythm, emphasizing the adaptability and ingenuity of the scientific community in understanding this vital physiological process.

• **Rhythm Generation** In the examination of respiratory rhythm generation, we observed a variety of methodological approaches across publications. Specifically, 11 publications exclusively utilized *in vitro* models, while one publication relied solely on *in vivo* models. Additionally, seven publications adopted a combined approach, utilizing both *in vivo* and *in vitro* models.

In the context of *in vivo* techniques, nine publications primarily employed whole-body plethysmography for the study of respiratory rhythm. In contrast, *in vitro* methodologies were diversified, with 15 publications relying on recordings from the hypoglossal or phrenic nerve as their primary means of assessing changes in respiratory rate. However, ten publications favored patch-clamp techniques, and six conducted extracellular recordings in the brainstem.

• **Rhythm Modulation** In the exploration of respiratory rhythm modulation, we identified a total of 52 publications, each with its unique approach. Of these, 22 publications exclusively employed *in vitro* methods, 23 opted for *in vivo* approaches, and seven adopted a combined approach, utilizing both *in vivo* and *in vitro* techniques.

Notably, the most prevalent technique for *in vivo* studies was whole-body plethysmography, with 31 publications utilizing this approach to investigate respiratory rhythm modulation. Conversely, in *in vitro* studies, the most commonly used techniques included recordings from the hypoglossal or phrenic nerve, employed in 22 publications, ten publications performed patch-clamp techniques, and seven publications conducted extracellular recordings in the brainstem.

• **Rhythm Generation and Modulation** Among publications focused on both rhythm generation and modulation, three exclusively employed *in vitro* approaches, two utilized *in vivo* techniques, and four publications adopted a combination of *in vivo* and *in vitro* methods.

Significantly, the dominant *in vivo* technique for studying respiration was whole-body plethysmography, featured in six publications. In contrast, the primary *in vitro* techniques used to analyze respiratory patterns included patch-clamp cell recording, conducted in two publications, recordings from the hypoglossal or phrenic nerve, used in two publications, and extracellular recordings in the brainstem, reported in one publication.

**Evaluation:** The analysis reveals a varied landscape of methodological approaches in the study of respiratory rhythm. Researchers have employed a diverse range of *in vivo* and *in vitro* techniques, with whole-body plethysmography and nerve recordings featuring prominently. The flexibility in method selection allows for a comprehensive investigation into both rhythm generation and modulation. These findings underscore the multidimensional nature of respiratory research and the importance of choosing appropriate methodologies to elucidate this intricate biological process.

#### 3.1.3 Electrophysiological preparation

The study of respiratory rhythm is marked by a diversity of electrophysiological preparations employed to investigate both rhythm generation and modulation. These distinct methodologies offer valuable insights into the complexity of this physiological process.

• **Rhythm Generation** The preparations used for electrophysiological recordings in the study of rhythm generation are diverse. Among these, the most commonly adopted approach is the use of brainstem-spinal cord (*en bloc*) preparations in eleven publications. Furthermore, researchers have utilized rhythmic medullary slices in nine publications, the working heart-brainstem preparation (WHB) in two publications, spinal cord preparations in one publication, organotypic cultures in one publication, and non-rhythmic slices with the pons preserved in one publication.
• **Rhythm Modulation** In the exploration of respiratory rhythm modulation, distinct preparations for electrophysiological recordings have been used. The most prevalent approach is the utilization of *en block* preparations, reported in 17 publications, followed by rhythmic medullary slices in 13 publications, the WHB preparation in 6 publications, spinal cord preparations in one publication, organotypic cultures in one publication, and non-rhythmic slices in one publication.
• **Rhythm Generation and Modulation** Regarding electrophysiological preparations for the study of both respiratory rhythm generation and modulation, the most common methods include the use of (*en bloc*) preparations, employed in three publications and rhythmic medullary slices reported in two publications.

**Evaluation:** Researchers often favor *en bloc* preparations and rhythmic medullary slices, aligning with their suitability for capturing and understanding the intricacies of respiratory patterns, primarily due to the presence of the pre-Bötzinger complex, the central pattern generator of respiratory rhythm. While these preparations are frequently favored due to their utility in capturing and exploring respiratory patterns, it is essential to consider other preparation methods for a comprehensive understanding of this intricate physiological process. The incorporation of alternative preparations can provide additional perspectives and contribute to a more integrative comprehension of respiratory rhythm.

#### 3.1.4 Age range of subjects in electrophysiological experiments

Understanding the intricacies of respiratory rhythm generation and modulation is not only contingent on precise experimental techniques but also on the age of the animals under investigation. In this section, we delve into the critical dimension of the age of the animals utilized for electrophysiological recordings.

• **Rhythm Generation** In the context of rhythm generation studies, the age of rodents employed exhibited substantial variation. Researchers utilized specimens spanning from embryonic day 9.5 (E9.5) to postnatal day 12 (P12). Furthermore, certain investigations extended their focus to include adult rodents aged between 10 and 21 weeks.
• **Rhythm Modulation** Studies dedicated to the exploration of rhythm modulation encompassed rodents at diverse stages of development. The subjects ranged from E9.5 to rodents up to P21. In addition, certain investigations expanded their scope to encompass adult rodents aged between 10 and 28 weeks.
• **Rhythm Generation and Modulation** For studies that delved into both rhythm generation and modulation, the age of rodents under examination similarly exhibited significant variability. Researchers incorporated specimens ranging from E14.5 to adult rodents aged 8–20 weeks.

**Evaluation:** Overall, the variations in the ages of rodents used across these studies highlight the multidimensional nature of respiratory rhythm research. These findings underscore the importance of taking an integrative approach, encompassing different stages of development, to comprehensively unravel the complexities of respiratory rhythm in transgenic rodents. Researchers in this field should be cognizant of the unique insights that can be gained by considering different developmental stages, contributing to a more nuanced understanding of respiratory rhythm regulation.

#### 3.1.5 Complementary techniques employed

The study of respiratory rhythm encompasses a wide array of techniques that extend beyond electrophysiology. While electrophysiological approaches provide critical insights into the neural and cellular mechanisms governing respiratory patterns, other techniques offer complementary perspectives on this intricate physiological process.

• **Rhythm Generation** An examination of respiratory rhythm generation across 19 publications revealed a diverse array of techniques. The most frequently employed method was immunohistochemical techniques in 12 publications, followed by seven that used electromyography, six that employed *in situ* hybridization, three that utilized calcium imaging, two that reported optogenetics, and one each that applied Western blot, used polymerase chain reaction (PCR), implemented chemogenetics, and employed electron microscopy.
• **Rhythm Modulation** Within publications dedicated to respiratory rhythm modulation, immunohistochemical techniques emerged as the predominant approach, with 24 publications employing this method. Additionally, a variety of other techniques were utilized, including pharmacology in ten publications, electromyography in four publications, optogenetics in five publications, Western blot in four publications, PCR in three publications, *in situ* hybridization in two publications, chemogenetics in two publications, and calcium imaging in two publications.
• **Rhythm Generation and Modulation** In publications addressing both respiratory rhythm generation and modulation, a variety of techniques were harnessed. The immunohistochemical technique was used in three publications, ELISA in two publications, calcium imaging in two publications, electromyography in one publication, pharmacology in one publication, and optogenetics in one publication.

**Evaluation:** The comprehensive analysis of techniques employed in the study of respiratory rhythm generation, modulation, and their interplay underscores the versatile nature of research in this field. The prevalence of immunohistochemical techniques suggests their utility in capturing the underlying mechanisms of respiratory rhythm. Additionally, the diversity of techniques used highlights the multidisciplinary approach necessary to gain a comprehensive understanding of respiratory control. Researchers have adeptly leveraged a range of methods to probe the intricacies of respiratory rhythm, enhancing the breadth and depth of knowledge in this vital area of physiology.

### 3.2 Potential transgenic mouse lines for studying respiratory rhythm generation and modulation: An in-depth analysis

The landscape of transgenic mouse lines offers a rich resource for studying the intricacies of respiratory rhythm generation and modulation. This section aims to provide a comprehensive review of relevant transgenic mouse lines, categorizing them based on their respiratory phenotypes, and further delves into their implications for both basic research and potential medical applications.

#### 3.2.1 Phenotypic variation across lines

We have identified a total of 119 distinct transgenic mouse lines displaying various respiratory rate abnormalities (Mouse Genome Informatics definitions are provided in parentheses), as shown in Supplementary Table S5. Here, we present a brief overview of these transgenic lines.

• 38 lines displayed an increased pulmonary respiratory rate (greater than the normal number of breaths per minute).
• 23 lines exhibited tachypnea (rapid breathing).
• 43 lines demonstrated a decreased pulmonary respiratory rate (fewer than the normal number of breaths per minute).
• 4 lines presented abnormal pulmonary respiratory rate (deviation from the normal number of breaths per minute).
• 1 line showed respiratory failure (cessation of or failure to commence breathing).

#### 3.2.2 Focus on preBötC

Within the preBötC, a crucial region for respiratory rhythm generation.

• 11 lines showed abnormal physiology (any functional anomaly of the group of interneurons within the medulla oblongata’s ventral respiratory group that are important for the generation of ventilatory (inspiratory) rhythmogenesis).
• 3 lines exhibited abnormal morphology (any structural anomaly of the group of interneurons within the medulla oblongata’s ventral respiratory group that are important for the generation of ventilatory (inspiratory) rhythmogenesis).
• 2 lines presented abnormal central pattern generator function (any functional anomaly of the neural networks that produce rhythmic patterned output without sensory input and underlie rhythmic motor patterns).

Evaluative Note: Although abundant, the existing literature lacks specification regarding the sex of mice used in 99 publications, posing a significant limitation to the generalizability of the findings. It is important to note that some transgenic lines exhibit more than one phenotype, which reflects the variability within transgenic lines but is also limited by the studies conducted on these lines. In some lines, central alterations have been examined, while in others, alterations have only been detected through plethysmography studies, suggesting potential changes in respiratory rhythm generation or modulation that have not been thoroughly explored.

#### 3.2.3 Relevance to human disease models

Significantly, 34 of the identified transgenic mouse lines serve as models for various human diseases such as acute myeloid leukemia, malignant hyperthermia, hypertrophic pyloric stenosis, glycine encephalopathy, among others.

#### 3.2.4 Unique and novel lines

Our investigation further led us to unique lines, such as a non-gene modification-induced bradypneic phenotype line (bd/bd). Notably, we identified two lines—Taar6 (Trace amine-associated receptor 6) and Scgb1a1 (Secretoglobin Family 1A Member 1 — whose expressed genes have not been previously reported in the context of the brainstem.

#### 3.2.5 Most frequently used lines

The most prevalent transgenic mouse line in existing publications is the Myeloproliferative neoplasm model (MGI:6356966), featuring the Jak2 (Janus kinase 2) gene. Although this model is known for tachypnea, its direct relevance to respiratory rhythm remains unexplored.

### 3.3 Bibliometric analysis

A bibliometric analysis is a quantitative study of research publications, comprising a range of techniques employed for the examination and quantification of texts and information, particularly within extensive datasets (Cobo et al., 2011). Conducting such an analysis on publications that employ transgenic rodents to investigate respiratory rhythm is highly relevant within the context of a scoping review. This approach provides valuable insights into the research landscape, revealing trends, influential contributors, and emerging areas of interest. This information could guide future research directions and resource allocation, ultimately advancing our comprehension of respiratory rhythm generation and modulation.

#### 3.3.1 Overview

The analysis unveiled that the 80 reviewed publications span the years from 1997 to 2022, with an average document age of 12.5 years, and originate from 30 different journals. The annual growth rate of publications investigating respiratory rhythm through the use of transgenic rodents stands at 5.7% per year. These publications collectively involved 424 researchers, resulting in an average of 6.9 co-authors per publication, and garnered an average of 49.76 citations per publication.

**FIGURE 3:**
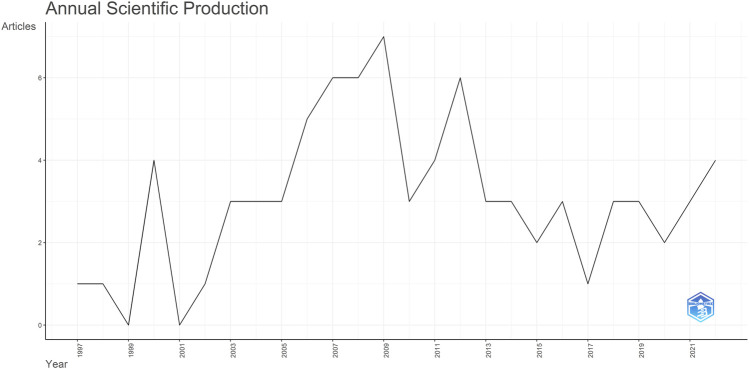
Yearly trends: Respiratory rhythm generation and modulation in transgenic rodents.

The annual scientific production shows an uptick in publications starting in 2002, peaking in 2009 and 2012 with 7 and 6 publications, respectively. However, research has waned since 2013; in the last 3 years, the annual publication count has fallen to just 3 (Figure 3). Notably, nearly half of the publications come from the following journals: Journal of Neuroscience (18 publications); Respiratory Physiology & Neurobiology (10 publications); Journal of Physiology (five publications); eLife (four publications); and European Journal of Neuroscience (four publications).

Regarding authorship by country, corresponding authors are primarily from the USA (30 publications), followed by France (19 publications), Germany (10 publications), Japan (eight publications), and Sweden (four publications). It is noteworthy that over 50% of the publications can be attributed to research conducted in the USA and France.

**FIGURE 4:**
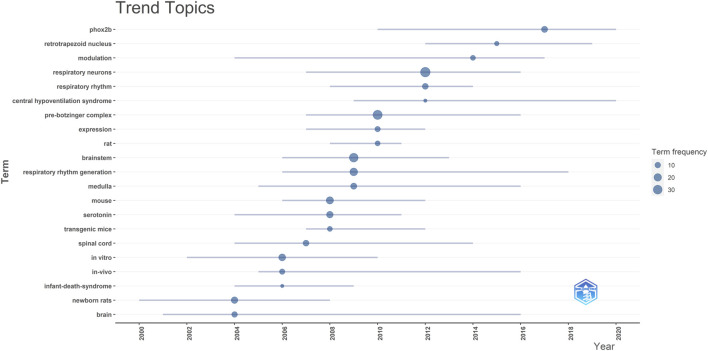
Illustrates the trend topics from the reviewed publications. Each line on the chart spans the years in which a specific topic was trending. The dot on each line pinpoints the year with the highest frequency of that topic’s mention.

The analysis indicates that the top ten most frequently occurring words in the reviewed publications, in descending order, are: “respiratory neurons,” “pre-Bötzinger complex,” “brainstem,” “respiratory rhythm generation,” “mouse,” “*in vitro*,” “newborn rats,” “serotonin,” “medulla,” and “phox2b” (see Figure 4). As this field evolves, each term exhibits a different trend over time, as depicted in Figure 4. Notably, terms like “rat” show a shorter period of prominence, while others like “brain” and “modulation” have consistently trended over time.

#### 3.3.2 Social structure

This section aims to analyze interactions and collaborations among researchers, organizations, and countries, identifying their organizational and collaborative patterns.

**FIGURE 5:**
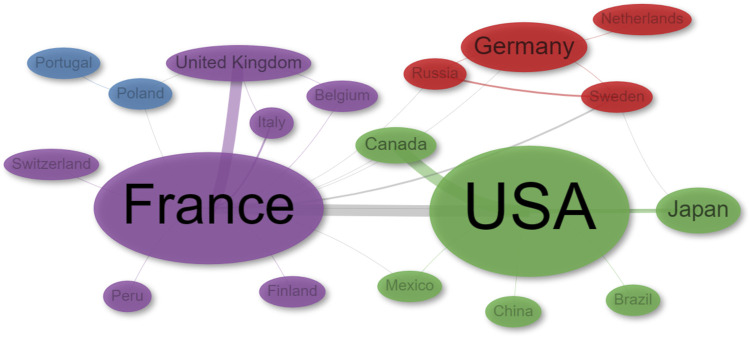
Network of collaborations by Author’s country of Affiliation.

In terms of international collaborations, the analysis identified four distinct clusters (Figure 5; Supplementary Table S1). The main clusters are the USA (green) and France (purple), and they also exhibit significant collaboration between them. The red cluster acts as a bridge between the green and purple clusters, with a notable involvement of Sweden. On the other hand, Poland and Portugal tend to collaborate closely with France, often in partnership with Belgium and Italy. Within the USA cluster, Canada is a key collaborator, while the UK plays a similar role within the France cluster (Figure 5).

**FIGURE 6:**
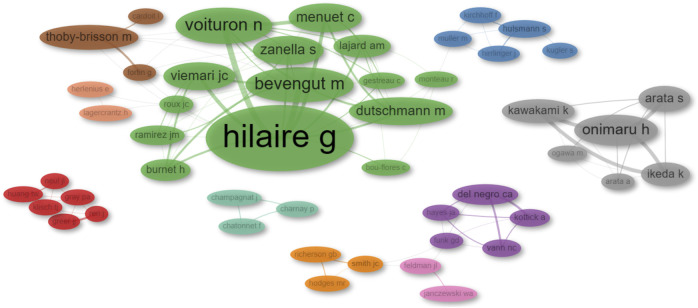
Network diagram illustrating author collaborations. The diameter of each dot corresponds to the total number of publications published by that author. The thickness of the connecting lines denotes the number of collaborative works between pairs of authors.

The collaboration network of authors (co-authorship network) represents collaborative relationships among researchers based on their joint authorship. Through network analysis, we identified ten distinct research groups. To avoid bias in naming clusters, we ranked them by PageRank score provided by the analysis. It is important to note that this ranking does not imply the author is the leader or the most relevant in their respective cluster, but rather that implies that an author is more likely to collaborate with these authors. The research groups identified were the following: Swen Hülsmann (red), Gérard Hilaire (blue), Christopher A. Del Negro (green), Jeffrey C. Smith (purple), Muriel Thoby-Brisson (orange), Jack L. Feldman (brown), John J. Greer (pink), Hiroshi Onimaru (gray), Jean Champagnat (light green), and Eric Herlenius (pink and orange). These results are illustrated in Figure 6; Supplementary Table S2.

This analysis has illuminated the presence of two major supergroups in collaboration: the Hilaire-Thoby-Brisson-Herlenius-Hülsmann group and the Del Negro-Smith-Feldman group. Additionally, our findings indicate the existence of three independent research clusters led by Onimaru, Champagnat, and Greer, respectively.

#### 3.3.3 Intellectual structure

To understand the fundamental concepts in this research field, we constructed a co-citation network, examining how often two documents are cited together. This analysis illuminates the foundational publications in the study of respiratory rhythm generation and modulation using transgenic rodents.

**FIGURE 7:**
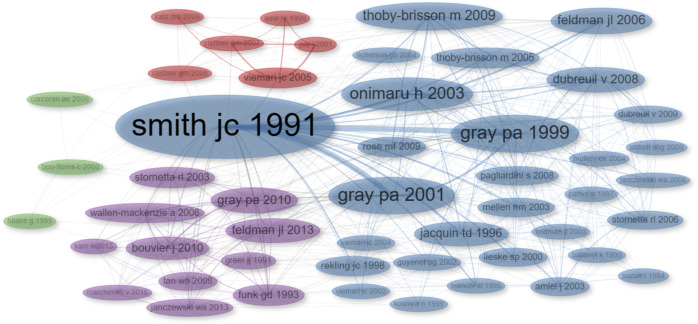
Co-citation Network Visualization. The size of each dot corresponds to the number of times the publication has been cited, and the thickness of the connecting lines indicates the frequency of co-citation between publications. Different dot colors indicate distinct clusters within the network.

Our analysis revealed four distinct clusters of references within the literature reviewed here (Figure 7; Supplementary Table S3). The largest cluster (blue) prominently features the work by Smith et al. (1991). The second cluster (purple) encompasses the contributions of Gray et al. (2010) and Feldman et al. (2013). These two clusters constitute the foundational literature on respiratory rhythm, covering aspects from genetics to neural circuits. The third cluster (red) centers around the research conducted by Viemari et al. (2005b) and pertains to investigations related to Rett syndrome. The fourth cluster (green) includes the works of Bou-Flores et al. (2000), Hilaire and Duron (1999), and Corcoran et al. (2009), which are associated with research on the role of serotonin.

#### 3.3.4 Conceptual structure

This section explores the conceptual relationships and intellectual foundations of this research field by analyzing the underlying themes, concepts, and knowledge organization within the literature.

**FIGURE 8:**
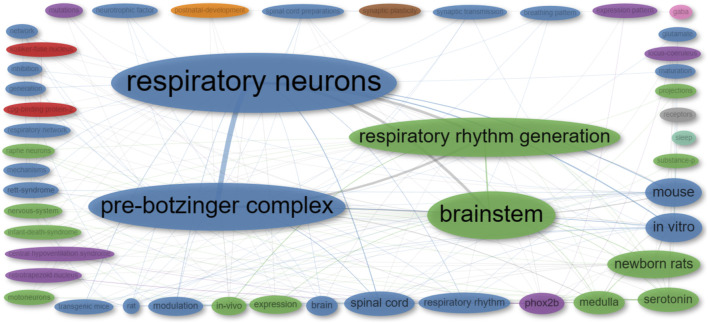
Co-occurrence network of ‘Keywords Plus’ from publications included in this review. The size of each dot corresponds to the frequency of the term in the publications, while the width of the lines indicates the frequency with which the two terms appear together.

To uncover the foundational concepts in the reviewed publications, we conducted a co-occurrence network analysis, which examines how often specific terms or concepts appear together, revealing associations and patterns. The analysis categorized the publications into nine clusters. Notably, five of these clusters are represented by a single term each (“post-natal development,” “synaptic plasticity,” “GABA,” “receptors,” and “sleep”), while one cluster is represented by two terms (“CPG-binding protein 2″ and “Kölliler-Fuse nucleus”). The primary cluster, denoted by the color blue, focuses on the neural control of respiratory rhythm. The green cluster is associated with the neurobiological aspects of respiratory rhythm generation and modulation. Lastly, the purple cluster is centered around Phox2b and its connection to central hypoventilation syndrome (Figure 8; Supplementary Table S4).

**FIGURE 9:**
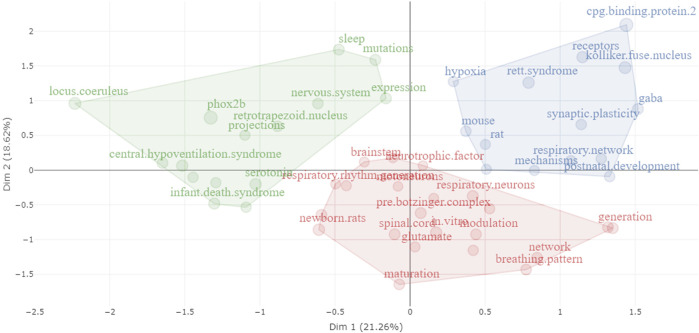
Visualization of the conceptual structure using Multiple Correspondence Analysis (MCA) on KeyWords Plus extracted from publications encompassed in this review. Distinct clusters are differentiated by unique colors, with terms associated with a particular cluster displayed in its respective color.

To unveil connections between research concepts and their co-occurrence patterns, we employed the multiple correspondence analysis method, which assists in visualizing and understanding complex relationships among categorical data. This analysis reveals that the reviewed publications can be categorized into three distinct groups (Figure 9).

• Central hypoventilation syndrome (Green Group): This group encompasses terms related to the central hypoventilation syndrome. These terms cover various aspects, including physiological factors (ventilatory response and sleep), genetic elements (Phox2b, expression, mutation, and expression pattern), and neural components (serotonin, locus coeruleus, raphe neurons, projections, retrotrapezoid nucleus, and substance P).
• Rett syndrome (Blue Group): The blue cluster centers around Rett syndrome, encompassing terms linked to animal models used to study the syndrome (rats and mice). It also involves alterations observed in Rett syndrome (inhibition, Kölliker-Fuse nucleus, synaptic plasticity, GABA receptors, hypoxia, respiratory rhythm, and post-natal development). These alterations are the result of mutations in the Mecp2 gene (i.e., CpG binding protein 2).
• preBötC (Red Group): The red cluster pertains to terms associated with the preBötC and the study of respiratory control. This includes a range of concepts, from anatomical aspects (respiratory neurons, preBötC, brainstem, medulla, and spinal cord) to physiological components (modulation, synaptic transmission, breathing patterns, and neurotrophic factors). Additionally, the cluster encompasses various study models (*in vitro* and *in vivo* studies) and the utilization of neonatal rats in research.

**FIGURE 10:**
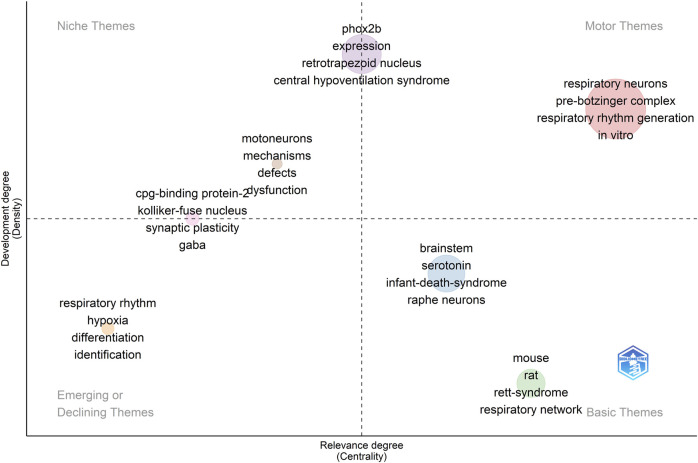
A thematic map illustrating the research domain of respiratory rhythm generation and modulation. The dot size indicates the frequency of KeyWords Plus in each cluster. The displayed terms are the four most common words within the cluster. Clusters of higher relevance to the field are oriented to the right, while more mature clusters are placed at the top.

We have also constructed a thematic map, or strategic diagram, to examine the principal themes and subjects related to the use of transgenic rodents in the context of studying respiratory rhythm generation and modulation (see Figure 10). These maps are a type of co-word analysis employed to identify clusters of keywords, which are referred to as themes. Each theme is characterized by two key parameters: density, which measures the strength of its external connections with other themes, signifying its significance in shaping the overall research domain, and centrality, which assesses the strength of internal connections among all keywords associated with that theme, reflecting the theme’s development Cobo et al. (2011). Consequently, this map allows us to pinpoint pivotal topics, comprehend their relationships, and highlight areas where further research is needed.

Within this map, several thematic categories emerge. Specifically, terms such as “respiratory neurons,” “pre-Bötzinger complex,” “respiratory rhythm generation,” and “*in vitro* research” are identified as core themes, often referred to as motor themes, representing the fundamental concepts in this field (see Figure 10). In contrast, terms like “motoneurons,” “mechanisms,” and “defects” fall into the category of niche themes, indicating specialized research areas (see Figure 10).

Furthermore, “Phox2b,” “expression,” “retrotrapezoid nucleus,” and “central hypoventilation syndrome” serve as both core and niche themes. We have also observed that certain themes such as “respiratory rhythm,” “hypoxia,” “differentiation,” and “identification” are either diminishing or emerging. These represent subjects that have experienced changes in their centrality and density within the research landscape over time (see Figure 10).

Lastly, our map identifies foundational themes such as “brainstem,” “serotonin,” and “infant death syndrome,” along with species-specific terms like “mouse” and “rat.” These are considered basic themes, representing the essential components of the broader research field (see Figure 10).

## 4 Discussion

Based on our review, we found three reviews regarding the use of transgenic rodents for studying respiratory rhythm: Katz and Balkowiec (1997); Williams and Burdakov (2008), and Gaultier and Gallego (2008). Additionally, Gaultier et al. (2006) and Gaultier et al. (2003) have presented two reviews that delve into transgenic models tailored for examining disorders associated with respiratory control and chemical breathing control, respectively. Notably, the modern research landscape lacks a scoping review on this topic, and the studies were published over 15 years ago.

In this scoping review, various transgenic rodent lines used in respiratory rhythm research are discussed. This includes 30 lines dedicated to rhythm generation, 61 lines targeting rhythm modulation, and 16 lines serving dual purposes, all of which are elaborated in Tables 1-3. Among these, it is imperative to highlight that two lines have been specifically developed for visualizing respiratory neurons: one tailored for rats as described by Ikeda et al. (2015) and another for mice as documented by Winter et al. (2007). In these studies, it is important to note that no additional experiments were conducted; rather, the focus was solely on the creation of new transgenic animals. These lines were designed to specifically identify neuronal populations within the respiratory CPGs. Consequently, they pave the way for a profound exploration of electrophysiological attributes and the dynamic responses to the activation or inhibition of distinct channels, receptors, or metabolic routes.

### 4.1 Techniques for targeted manipulation in transgenic rodents

While the primary method for studying respiratory rhythm involves electrophysiological or plethysmography recording, the utilization of transgenic rodents offers the advantage of employing additional techniques like optogenetics, chemogenetics, and viral transduction. These methods enable selective activation or inhibition of specific cell types, even in awake and conscious animals, thereby allowing for the evaluation of the role of these cells within the entire respiratory system. This approach preserves all the mechanisms associated with respiratory rhythm generation and modulation. In the following paragraphs, we summarize the publication using these techniques.

#### 4.1.1 Optogenetics

Within the realm of optogenetics applied to transgenic rodent models, this review identified eight pivotal publications that harness this technique (Kottick and Del Negro, 2015; Cui et al., 2016; Koizumi et al., 2016; Vann et al., 2016; Ausborn et al., 2018; Vann et al., 2018; Ikeda et al., 2019; Huff et al., 2022). These investigations predominantly utilized transcription factors, namely, Phox2b, Dbx1, and Sst, as distinctive markers for the formulation of these models. It is important to emphasize that only the study by Ausborn et al. (2018) incorporated transgenic mice with the expression of channelrhodopsin-2, guided by the VGAT-promoter, without confining its expression strictly to the preBötC domain. Furthermore, among this collection of studies, Ikeda et al. (2019) remains unique in its exploration using transgenic rats.

Collectively, the aforementioned studies corroborate the pivotal role of distinct neurons located within the respiratory CPGs in both the generation and modulation of respiratory rhythm. To illustrate, Cui et al. (2016) adeptly employed this methodology to discern a microcircuit within the preBötC dedicated to rhythm generation, alongside another for pattern generation. Complementing this, both Cui et al. (2016) and Ausborn et al. (2018) illuminated the existence of an inhibitory post-inspiratory neuronal population situated within the preBötC. In further elucidation, Ausborn et al. (2018) delineated the intricate interplay between the preBötC and BötC, signifying their combined influence in sculpting the respiratory cycle.

Distinct neuronal activations have demonstrated specific effects on respiratory patterns. For instance, when Phox2b neurons were activated, there was a marked reduction in the respiratory rate, as highlighted by Ikeda et al. (2019). In a contrasting observation, the photoinhibition of Dbx2 neurons precipitated a decrease or even cessation of breathing in adult mice, as documented by Vann et al. (2016) and Vann et al. (2018). Moreover, the photoactivation of Dbx1 neurons was found to induce short-term depression, which had a profound effect on the inspiratory-expiratory transition—a pivotal mechanism in respiratory rhythm generation (Kottick and Del Negro, 2015).

In a nuanced exploration, Koizumi et al. (2016) introduced three triple-transgenic mouse lines, shedding light on the shared voltage-dependent frequency control property among glutamatergic, Dbx1-derived, and Sst-expressing neurons within the preBötC. This revelation underscored that these neurons, in their collective capacity, form a singular excitatory population instrumental in the respiratory rhythm generation. Augmenting this body of knowledge, Huff et al. (2022) posited that the preBötC, beyond its foundational role in respiratory rhythm generation, also plays an integral part in orchestrating the nuances of breathing and its shallowing dynamics.

In summary, the utilization of optogenetics in transgenic rodent models within these studies has significantly advanced our comprehension of respiratory central pattern generators. These investigations highlight the pivotal role played by specific neuronal populations in respiratory rhythm generation and modulation. Noteworthy disparities in transgenic models and distinctive optogenetic methodologies have unveiled the intricate microcircuits inherent in the preBötC. Additionally, they have highlighted the collaborative interplay between the preBötC and BötC in the shaping of the respiratory cycle. The findings manifest the unequivocal influence of specific neuronal activation on respiratory patterns. Despite the inherent limitations of optogenetics, these inquiries contribute substantially to our understanding of respiratory control. Nevertheless, it is imperative to be aware of the fact that, for example, variations in stimulation parameters, even when specifically targeting the same cerebral region, emanate from the inherent intricacies of neural circuits. Neurons may exhibit diverse responses to distinct stimulation conditions, necessitating adjustments contingent upon the selection of optogenetic tools and transgenic models. This diversity in experimental configurations underscores the dynamic nature of neural systems and the significance of customized approaches in the context of optogenetic studies involving transgenic rodent models.

#### 4.1.2 Viral transduction

Viral vectors have emerged as invaluable tools in neuroscience, offering a powerful alternative to traditional transgenic rodent models. While traditional transgenic rodents involve the permanent integration of genetic material into the genomes of virtually all cell types, viral vectors provide a dynamic, temporary, and precise means of delivering genetic material to specific cell populations. Notably, viral vectors are not only a powerful standalone approach but also highly compatible with transgenic animals, enhancing their value. This compatibility allows for an even more versatile and refined exploration of neural circuits and expands their potential applications.

In this review, we identified nine publications using viral vectors to target specific neurons and explore their function. In Fu et al. (2019) AAV2 vectors were used to introduce the human M3 muscarinic receptor into NTS neurons of Phox2b-cre mice, providing unequivocal evidence of a neuroanatomical and functional projection from these neurons to the preBötC, which is involved in potentiating baseline pulmonary ventilation. In a similar manner, Tan et al. (2008) used AAV2 to express allatostatin receptors in somatostatin-expressing neurons of the preBötC, demonstrating the necessity of these neurons for breathing in adult awake rats. Notably, their work achieved transduction in a non-transgenic rat. In their study Vann et al. (2018), AAV was injected to induce Flp-mediated recombination of Frt sites, restricting the expression of CatCh (a Ca^2+^ translocating channelrhodopsin) to Dbx1 neurons in adult Dbx1; CatCh mice. This experiment confirmed that these neurons, putatively the preBötC core, serve as the rhythmogenic core responsible for respiratory rhythm generation and modulation, similar to neonatal animals. In their experiment, Cui et al. (2016) Sst-neurons in the preBötC of Sst-cre mice were targeted using AAV (AAV2/1-Ef1*α*DIO-ChR2-eYFP). This revealed that Sst-neurons in the preBötC are not a single population. Instead, there is a group that contributes to the pattern generation of the inspiratory burst and another group that plays an inhibitory role within the preBötC. As part of their investigation, Souza et al. (2020) achieved specific transduction of Phox2b-neurons in the NTS (using LVV-PRSx8-ChR2(H134R)-mCherry and rAAV5-Flex-taCaspase3-Tevp) and C1 neurons in TH-cre rats (with rAAV2-eF1*α*-DIO-hChR2(H134R)-eYFP). This approach allowed them to determine the distinct roles of these closely located populations in active inspiration and arousal. Fortuna et al. (2019) explored AAV’s potential to transduce glycinergic neurons in the Bötzinger and pre-Bötzinger Complex, showcasing precision in their injections in mice. In contrast to the aforementioned studies, Mironov et al. (2009) utilized AAV to transfect a calcium sensor (D3cpv) into neurons in an organotypic slice from a Rett syndrome mouse model. This approach revealed that these neurons exhibit deregulated calcium buffering. Conversely, Quintana et al. (2012) and Matagne et al. (2021) employed viral vectors to create and assess gene therapies for Rett syndrome and Leigh syndrome, respectively.

Collectively, these studies underscore the significant role of viral vectors in advancing our understanding of respiratory rhythm generation and modulation. They have also demonstrated the potential of these vectors in the development of gene therapies for diseases impacting respiratory rhythm. Viral vectors offer versatility and precision, expanding the horizons of neuroscience research. Nevertheless, it is crucial to recognize their limitations, such as tropism, the potential for evoking immune responses in the host organism, as well as challenges related to consistency and effectiveness.

#### 4.1.3 Chemogenetics

Chemogenetics has become a powerful technique for studying respiratory rhythm generation and modulation in transgenic rodents. By utilizing Designer Receptors Exclusively Activated by Designer Drugs (DREADDs) or other receptors whose ligands are not naturally present in the system, researchers can precisely control neuronal activity. This dynamic tool enables the investigation of neural circuits, including those responsible for respiratory function, offering advantages such as temporal flexibility and cell-type specificity. This approach allows for a more in-depth exploration of respiratory central pattern generators and their role in shaping breathing patterns. Consequently, chemogenetics opens new avenues for understanding the intricacies of respiratory control in transgenic rodent models.

Although limited, in this review we identified 3 publications reporting the use of chemogenetics. In the study by Tan et al. (2008), the expression of the allatostatin receptor, a G protein-coupled receptor found in *Drosophila*, was employed to rapidly and reversibly inactivate neurons by opening K^+^ channels. This approach enabled the transient silencing of Sst-neurons in the preBötC of awake rats. Forsberg et al. (2017) focused on expressing the MrgA1 receptor, a normally non-brain-expressed Gq-coupled receptor. Through this method, they activated astrocytes in organotypic slices of the preBötC and pFRG/RTN, revealing the release of prostaglandin E2 by astrocytes in response to hypercapnic conditions. Lastly, Fu et al. (2019) induced the expression of the human M3 muscarinic receptor (hM3Dq), a Gq-coupled receptor, in the NTS. This allowed for the activation of Phox2b-expressing NTS neurons in conscious animals, ultimately demonstrating the necessity of these neurons in the hypercapnic ventilatory response.

These studies collectively exemplify the power of chemogenetics in unraveling the complexities of neural circuits involved in respiratory rhythm generation and modulation. This approach has allowed researchers to make significant strides in understanding the roles of Sst-neurons, astrocytes, and Phox2b-expressing NTS neurons in respiratory control. While research using chemogenetics in transgenic rodents for studying respiratory rhythm generation and modulation is still relatively limited, these studies emphasize its potential to broaden our understanding of respiratory physiology.

As demonstrated by these studies, the utilization of transgenic rodents alongside optogenetics, chemogenetics, and viral transduction extends the toolbox for investigating respiratory rhythm, encompassing both whole-animal and system reduction approaches, such as electrophysiological preparations. Nevertheless, it is crucial to refrain from considering these techniques as infallible tools. As briefly outlined in this section, each of these methods offers distinct advantages and limitations. However, the scope of this review does not allow for an in-depth examination of these aspects. Thus, there is a need for future systematic reviews or meta-analyses centered on these techniques to enhance their precision, reliability, and consistency. Ultimately, this endeavor will contribute to the advancement of respiratory rhythm research.

### 4.2 Transgenic rodents as disease models for the study of respiratory rhythm generation and modulation

Factorial analysis, as delineated in Figure 9, accentuates the central themes in the utilization of transgenic rodents for exploring respiratory rhythm generation and modulation. Three principal categories emerge from this analysis: the preBötC (illustrated in red), Rett syndrome (portrayed in blue), and central hypoventilation syndrome (distinguished in green). This analytical perspective underscores that the overarching objective of deploying transgenic rodents in this domain is to unravel the intricate mechanisms orchestrating respiratory rhythm. Concurrently, these rodent models serve as indispensable tools, elucidating the ramifications of mutations in the *Mecp2* and *Phox2b* genes, especially in the manifestation of conditions like Rett syndrome and central hypoventilation syndrome.

#### 4.2.1 Rodent models of Rett syndrome

The implications of these investigations are profound, considering that an estimated 65% of the global Rett syndrome patient population experiences respiratory issues (Ramirez et al., 2013; Anderson et al., 2014). Our review led to the identification of 12 pivotal publications that leverage transgenic mouse models to study Rett syndrome, offering insights into the intricate mechanisms governing respiratory rhythm generation and modulation.

A case in point is the research by Schmid et al. (2012), which elucidated the instrumental role of BDNF-TrkB signaling in modulating respiratory frequency. By introducing a TrkB agonist, the researchers successfully reinstated the respiratory frequency in heterozygous female subjects to benchmarks akin to their wild-type counterparts. Similarly, Mironov et al. (2009) highlighted that introducing BDNF in *Mecp2* ^−/*y*^ mice reinstated the slow calcium buffering in preBötC neurons. This intervention also brought to light the nuanced alterations in the morphology and organization of these neurons. The authors postulate that the compromised calcium buffering might instigate a retraction in neuronal processes, thereby potentially reconfiguring the respiratory network.

A corollary to this observation is the assertion by Stettner et al. (2007) that such neural remodeling might permeate to the post-inspiration phase, intensifying the instances of apnea. Augmenting this perspective, mutations in *Mecp2* have been correlated with a diminution in TH-neurons, norepinephrine, and serotonin concentrations, introducing potential aberrations in the modulation of the respiratory network (Viemari et al., 2005b).

Kron et al. (2011) conducted a study wherein they discerned that hypoxia elicited depressive responses, which could potentially culminate in the cessation of preBötC activity, manifesting as apneic episodes. Interestingly, this adverse outcome could be circumvented through the introduction of a 5-HT_1*A*_ receptor agonist. In addition, the mice under study exhibited a marked excitatory/inhibitory disequilibrium. This was typified by a diminished GABAergic transmission and concomitant downregulation in the expression of VGAT and GABA_*A*_ subunits *α*_2_ and *α*_4_ (Medrihan et al., 2008).

These studies emphasize the utility of transgenic rodent models of Rett syndrome, which have elucidated mechanisms related to respiratory rhythm generation and modulation. These studies have explored a wide range of topics, including circuit maintenance and neurotransmission, using pharmacological approaches. While the primary aim is to develop potential pharmacological therapies, these studies also provide valuable insights into the essential elements for optimal functioning from a basic science perspective.

#### 4.2.2 Other rodent models of human diseases

Although Rett syndrome remains the focal point in investigations of respiratory rhythm alterations using transgenic models, other pathologies like Alzheimer’s disease (Menuet et al., 2011; Irmler et al., 2012), Pompe disease (DeRuisseau et al., 2009), Leigh syndrome (Quintana et al., 2012), multiple system atrophy (Flabeau et al., 2014), and amyotrophic lateral sclerosis (Lladó et al., 2006), have been probed as well.

DeRuisseau et al. (2009) showed that Pompe disease, typically characterized by pronounced muscular atrophy and resultant respiratory challenges, has a central component. Mice deficient in acid *α*-glucosidase (GAA^−/−^) displayed diminished breathing frequency and enlarged phrenic cell bodies, underscoring the indispensability of GAA for optimal respiratory functioning.

Conversely, studies by Quintana et al. (2012) and Irmler et al. (2012) highlighted respiratory rhythm disturbances in mice with NADH dehydrogenase [ubiquinone] iron-sulfur protein 4 (NDUFS4) deficiency or a mutated ubiquitin B (UBB^+1^) variant, respectively. The former revealed mitochondrial impairments in the dorsal brainstem vestibular nucleus of Ndufs4-KO mice, accompanied by perturbed preBötC activity. The latter demonstrated an accumulation of UBB in several areas of the brainstem that are essential for respiratory modulation, including the dorsal respiratory group, pneumotaxic center, and nucleus of the tractus solitarius, among others.

Menuet et al. (2011) explored the intersection between Alzheimer’s disease, tauopathies, and respiratory alterations, pinpointing serotoninergic modulation’s influence on preBötC functionality. Utilizing transgenic mice expressing a mutant form of the Tau protein (Tau.P301L), significant tauopathy was observed in the Kölliker-Fuse, raphé obscurus, and raphé magnus nuclei. Notably, medullary respiratory-associated regions were unaffected. This study hypothesized that disruptions in serotonin metabolism might perturb respiratory network modulation.

Parallel findings emerge from other transgenic models. Bou-Flores and Hilaire (2000) and Bou-Flores et al. (2000) manipulated serotoninergic metabolism by inactivating the monoamine oxidase A gene (MAO-A). In another approach (Hodges et al., 2009), and Corcoran et al. (2014) suppressed serotoninergic neuron formation by genetically deleting the transcription factor Lmx1b in Pet-1-expressing cells. Both approaches underscore the importance of serotonin in respiratory rhythm modulation. In a related aspect, Flabeau et al. (2014) revealed that transgenic mice expressing human wild-type *α*-synuclein in oligodendrocytes (PLP-*α*SYN) manifested enhanced respiratory variability and serotonin depletion relative to their wild-type counterparts, suggesting a regulatory role for serotonin in respiratory modulation.

Altogether, these studies highlight the significance of employing transgenic rodents as invaluable models for investigating respiratory rhythm generation and modulation in the context of various diseases. They not only enhance our comprehension of the underlying mechanisms but also shed light on the pathophysiology of conditions characterized by abnormal respiratory function. Additionally, transgenic rodent models provide a promising platform for the development of pharmacological and genetic strategies aimed at preventing or treating patients affected by these diseases. Therefore, it is essential to maintain a steadfast commitment to ongoing research utilizing these disease models.

### 4.3 Advantages, limitations, and suggestion for the use of transgenic rodent models to study respiratory rhythm generation and modulation

Transgenic rodent models, like other genetic models, can exhibit compensatory mechanisms that obscure the influence of a particular gene or cell type. However, these models have been instrumental in elucidating core biological processes encompassing reproduction, metabolism, feeding, and others. Currently, they play a pivotal role in unraveling a fundamental and complex mechanism shared by nearly all vertebrates, i.e., respiration. Despite the essential importance of breathing, its underlying mechanisms remained mysterious for an extended period.

This scoping review provided a comprehensive overview of the diverse array of transgenic rodent lines used in the study of respiratory rhythm generation and modulation (please refer to Section 4.4). Our examination of the literature reveals that the objectives for employing transgenic rodents are wide-ranging, spanning from investigating the developmental origins of respiratory neurons to manipulating neuronal or glial functions within CPGs and associated modulatory afferents. Furthermore, we have observed that the term “transgenic rodent” encompasses a broad spectrum of models. As detailed in Tables 1,2, and 3, transgenic lines can be categorized as either knockout or knock-in, based on whether they were used to inhibit (knockout) or introduce additional genetic material (knock-in), such as transgenes, to express or overexpress specific genes.

However, the field of genetic engineering has rapidly advanced, resulting in the development of more specialized transgenic lines that offer the advantage of spatial and timely inducibility, even with cell-specific precision. This evolution has furnished researchers with a highly valuable set of tools to test and validate hypotheses that would have been challenging to explore using traditional approaches.

#### 4.3.1 Advances in understanding respiratory rhythm

A seminal discovery made by Smith et al. (1991) pinpointed the CPG accountable for breathing, termed the pre-Bötzinger complex. Initially, the scientific consensus posited that the preBötC, akin to other CPGs, was populated by pacemaker neurons, a claim supported by Smith et al. (1991). At that juncture, the dominant supposition was that respiratory rhythm generation bore similarities to other recognized rhythmic processes. Yet, subsequent investigations revealed a more intricate landscape.

Over the course of the research, two supplementary CPGs, PiCo and pF_*L*_, were identified. These discoveries pivoted the emphasis from solely studying how the preBötC facilitates breathing to examining the collaborative function of these three CPGs in orchestrating respiratory rhythm. Concurrently, evidence emerged indicating that although preBötC pacemaker neurons were indispensable for initiating breathing, they alone were not sufficient (Del Negro et al., 2002). Contemporary hypotheses now posit that the preBötC is both necessary and sufficient for initiating respiratory rhythm, with its neuronal activity presenting as an emergent property (Del Negro et al., 2002).

Given its role as a CPG, the preBötC is inherently responsible for establishing both the rhythm and pattern of the inspiratory phase of respiration. This led to the question if a single neuronal population was responsible for both rhythm and pattern generation, or if there were specialized subpopulations. If such subpopulations were present, how did they synchronize to both produce and regulate the inspiratory phase and by extension, the entire respiratory cycle? This line of inquiry was pursued by multiple research factions.

Through our review, we observed that certain studies using transgenic rodents to investigate respiratory rhythm generation and modulation aimed to trace cell populations within the central pattern generators (e.g., Shirasawa et al., 2000; Blanchi et al., 2003) and their modulatory inputs (e.g., Dubreuil et al., 2009; Souza et al., 2020). These investigations have significantly contributed to our understanding of how alterations in their development could impact their function in adulthood. Furthermore, these studies have validated findings obtained through electrophysiological and pharmacological approaches, particularly in neonatal brainstem slices. An advantage of using transgenic rodents is the ability to conduct such experiments in conscious adult animals (e.g., Meier et al., 2007; Tan et al., 2008). In addition, the development of viral vectors has enabled researchers to achieve temporal and reversible control, along with target specificity, which has greatly facilitated the disentanglement of the intrinsic complexity of the neural network that controls the respiratory rhythm. With the concurrent development of optogenetics and chemogenetics, we are now poised to address some of the most fundamental questions regarding respiratory rhythm generation and modulation.

An example of the latter can be found in the investigation by Cui et al. (2016). As a preamble, Kam et al. (2013) employed an ingenious methodology involving the modulation of extracellular K^+^ levels. This approach allowed them to identify a pre-inspiratory population (pre-I) responsible for rhythmogenesis. The activity of this pre-I group serves as a precursor to the pattern formation executed by the inspiratory burst group (I). Building upon this work, Cui et al. (2016) utilized transgenic mouse strains (Dbx1-Cre; ChR2(H134R)-tdTomato and Sst-IRES-Cre; ChR2(H13R)-tdTomato) and transduction of Sst-cre mice with AAV2/1-EF1*α*-ChR2-eYFP into the preBötC, in combination with optogenetic techniques, to corroborate these observations *in vivo*. Their research further delineated a subsequent group known as the post-inspiratory population (post-I), which comprises GABAergic and/or glycinergic neurons.

#### 4.3.2 Phenotype variability

The preBötC functions as a CPG and, while it can autonomously generate respiratory rhythm and pattern, it also receives synaptic inputs. These inputs modulate the respiratory cycle to optimize parameters such as CO_2_/O_2_ levels, pH balance, temperature, and metabolic balance. Nonetheless, we should exercise caution when attributing the role of genes or cell types, as many transgenic models use genes primarily as tools to target specific cell types. Various genes and cell types often play roles in modulating and generating respiratory rhythm. It is crucial to note that not every gene or cell type associated with modulation or generation has the same impact.

Specifically, the genes *GLS1*, *KCC2a*, *kreisler*, *Lmx1b*, *Math1*, *Mecp2*, *PACAP*, *Phox2b-Atoh1*, *Atoh1*, *Jmjd3*, *Mafb*, *Rnx*, *STIM1*, *Tshz3*, *Vglut2*, and *UBB* have been observed to induce apneic episodes upon manipulation. In contrast. *H1R*, *L9′A*, *Mecp2*, *Nurr1*, *Phox2b*, *SLc6a5*, *MAOA*, *Atoh1*, *Rnx*, and *UBB*, result in tachypnea. The genes, *AE3*, *β* *-ENaC*, *DMPK*, *Gaa*, *KCC2a*, *Kreisler*, *Lmx1b*, *Math1*, *Mecp2*, *Nurr1*, *PACAP*, *Pet-1*, *Slc6a5*, *Tac1*, *Tau*, *chx10*, *Dbx1*, *Lbx1*, *Mafb*, *MAOA*, *ZFHX*, and *UBB*, when manipulated, lead to bradypnea. Additionally, *Kreisler*, *TH*, and *Lmx1b* are linked to polypnea; whereas *MAOA* is associated with atypical respiratory frequency. Some genes, including *A* *β* *PP*, *Epo*, *GDNF*, *Kir4.1*, *Mecp2*, *NMDAR1*, *Ret*, *Tacr1*, *Vgat*, and *α* *SYN*, do not influence this parameter. Notably, *ZFHX*, *Dbx1*, *ATP1a2*, *Tshz*, *Jmjd3*, and *Vglut2* have been implicated in causing respiratory failure or death. In terms of respiratory patterns, manipulation of *GDNF*, *KCC2a*, *Tacr1*, and *A* *β* *PP* appear to leave the pattern unaltered. Conversely, *Mecp2*, *MAOA*, *Phox2b-Atoh1*, *STIM1*, and *Mafb* cause irregularities, and *Tac1* demonstrates a unique breathing pattern, especially under hypoxic conditions.

#### 4.3.3 Consideration for comparative analyses

The varied phenotypes highlight the complex interactions and mechanisms within the respiratory network that influence rhythm and pattern. Notably, there lack of a uniform protocol for evaluating respiratory function in transgenic lines. This makes comparisons of genes or cell types impacts on the respiratory network challenging, particularly when specific genes are ubiquitously expressed across all tissues. Of the reviewed studies, 26 adopted an exclusive *in vivo* approach, predominantly leveraging plethysmography techniques to gauge the system’s integrative function. While this *in vivo* approach sheds light on the system as a whole, it may not precisely identify the locus of modifications. Conversely, 35 studies relied solely on *in vitro* preparations. These preparations offer detailed data about particular subpopulations or nuclei but might oversimplify the system, omitting crucial interactions. A combined *in vivo* and *in vitro* approach, as evidenced in 18 of the reviewed studies, provides the most thorough understanding, yielding both complementary and exhaustive insights into gene-specific mechanisms. The selected methodology should consistently reflect the research hypothesis.

In our examination of *in vitro* methodologies, we discerned four primary techniques for analyzing respiratory generation and modulation: working-heart brainstem (WHB; referenced in 32 publications), rhythmic medullary slice (cited in 25 publications), brainstem-spinal cord (*en bloc*; mentioned in 33 publications), and organotypic culture (referenced in 2 publications). There were also two publications that incorporated a spinal cord preparation, and another two that adopted a non-rhythmic slice, akin to the rhythmic medullary slice but with the preservation of the pons. Among these methodologies, the WHB approach (Paton, 1996) stands out as the most comprehensive. It allows for the examination of not only the CPGs but also other essential components of the respiratory network. On the other hand, the brainstem-spinal cord/*en bloc* (Smith and Feldman, 1987) and rhythmic medullary slice (Smith et al., 1991) techniques are predominantly centered on the preBötC and the relay of information to motor outputs, such as the hypoglossal and/or phrenic nerve. The organotypic culture method offers the distinct advantage of extended protocols for investigating the preBötC, as demonstrated by Rigatto et al. (2001) and Phillips et al. (2016). Nonetheless, the WHB preparation is particularly well-suited for *in vitro* studies, given its ability to maintain the integrity of the respiratory system. While it may lack sensory input and, consequently, the mechanisms of feedback and feedforward that are typically explored in *in vivo* approaches (e.g., whole-body plethysmography), it provides valuable insights into the central mechanisms governing breathing. It is worth noting that this preparation has even been employed for studying respiratory rhythm in adults (e.g., Dhingra et al., 2013; Koizumi et al., 2016), which is unusual as central pattern generators (CPGs) are typically studied in neonatal animals.

### 4.4 Limitations of this review and future work

While this review is comprehensive, it is essential to acknowledge its limitations. These limitations encompass the exclusion of non-English publications and the focus on specific databases. Despite the extensive research conducted, there remain unresolved questions. These pertain to conflicts in findings concerning the function of specific genes, methodological parameters in optogenetics, chemogenetics, and viral transduction, as well as the advantages and disadvantages of *in vitro* preparation. These questions, though highly pertinent, fall beyond the scope of this scoping review. Future research endeavors should concentrate on addressing these gaps, and the incorporation of a meta-analysis could provide a consolidated perspective on these matters.

Although this review gathered 80 publications, we identified that certain publications were not included in this scoping review, such as those by Sherman et al. (2015); Li et al. (2016); Graß et al. (2004); Oke et al. (2018); Baertsch and Ramirez (2019); Hirrlinger et al. (2019), and Oke et al. (2023). All of these publications, and potentially others, should have been included because they utilize transgenic rodents for studying respiratory rhythm generation and modulation. However, in some cases, they were not categorized with their corresponding MeSH terms. For instance, Li et al. (2016) employed two transgenic mouse lines (Nmbr^−/−^ and Grpr^−/−^), but it was not indexed with the MeSH term “Mice, Transgenic.” Similarly, Graß et al. (2004) conducted experiments in the respiratory network of transgenic mice (TgN (GFAP-EGFP)), but it was not associated with the MeSH term “Respiratory Center.” Remarkably, the publications by Oke et al. (2018) and Hirrlinger et al. (2019) in PubMed lacks MeSH terms altogether.

The constraints associated with MeSH terms have a substantial impact on our search strategy. MeSH terms play a crucial role in precision search, particularly in the context of well-established concepts, as they encompass synonyms and alternative spellings (Richter and Austin, 2012). However, it is crucial to acknowledge the limitations associated with MeSH terms, such as their inadequacy for capturing emerging concepts, the temporal lag in indexing, and incomplete coverage (Wang et al., 2022). These factors collectively contribute to the potential omission of relevant studies from our analysis. In light of these considerations, we acknowledge the limitations associated with MeSH terms and recognize the need for a more comprehensive search strategy. This involves combining MeSH terms and keywords, either as synonyms or to include terms not yet recognized by MeSH. A similar approach has been proposed to provide evidence for clinical questions (Ho et al., 2016).

## 5 Conclusion

The use of transgenic rodent models has been instrumental in advancing our understanding of respiratory rhythm generation and modulation. Key studies have emphasized the pivotal role of genes and their influence on the respiratory central pattern generators, notably the pre-Bötzinger complex. With insights spanning from the direct effects of specific genes on respiratory patterns to the methodologies adopted in studying these mechanisms, we recognize the profound implications of these findings for various respiratory ailments. While transgenic models have proved invaluable in deciphering core biological processes, they also present challenges, including potential compensatory mechanisms that might obscure genuine gene effects. As we move forward, it is imperative to adopt a combined *in vivo* and *in vitro* approach, ensuring a comprehensive understanding of respiratory rhythm. The intricate interplay of various genes and their role in respiratory rhythm not only deepens our grasp of the inherent nature of breathing but also offers a foundation for potential therapeutic strategies in respiratory diseases.

## Data Availability

The original contributions presented in the study are included in the article/Supplementary Material, further inquiries can be directed to the corresponding authors.
