# Supplementary material for: Transgenic rodents as dynamic models for the study of respiratory rhythm generation and modulation: a scoping review and a bibliometric analysis
Source: Front Physiol. 2023 Dec 21;14:1295632. doi: 10.3389/fphys.2023.1295632 (PMC10764557; doi:10.3389/fphys.2023.1295632)
Supplement: Supplementary file 1 [file DataSheet1.PDF]

Supplementary Material

1 SUPPLEMENTARY TABLES

Table S1. Country collaboration network analysis results

Country	Cluster	Betweenness	Closeness	PageRank
Germany	1	17	0.02857143	0.05125704
Sweden	1	1.66666667	0.02857143	0.0640145
Russia	1	0	0.02777778	0.04556623
Netherlands	1	0	0.01923077	0.01878686
Poland	2	17	0.02777778	0.04231433
Portugal	2	0	0.01886792	0.0198838
USA	3	35.5208333	0.03125	0.15594835
Japan	3	0.69230769	0.02857143	0.05045965
China	3	0	0.02040816	0.01617949
Canada	3	0	0.02777778	0.05796045
Brazil	3	0	0.02040816	0.01617949
Mexico	3	0	0.02777778	0.02482142
France	4	122.120192	0.04545455	0.24400736
Italy	4	0	0.02631579	0.03405503
United Kingdom	4	2	0.02857143	0.08354292
Switzerland	4	0	0.02564103	0.01653666
Belgium	4	0	0.02631579	0.0254131
Finland	4	0	0.02564103	0.01653666
Peru	4	0	0.02564103	0.01653666

Table S2. Author collaboration network analysis results

Author	Cluster	Betweenness	Closeness	PageRank
Greer JJ	1	0	0.14285714	0.02132421
Ren J	1	0	0.14285714	0.02132421
Gray pa	1	2	0.2	0.02172835
Huang TW	1	0	0.14285714	0.01817193
Klisch TJ	1	2	0.2	0.02172835
Neul JL	1	0	0.14285714	0.01817193
Hulsmann S	2	22	0.01333333	0.0282613
Hirrlinger J	2	0	0.01315789	0.01848152
Kirchhoff F	2	0	0.01315789	0.01848152
Kugler S	2	0	0.01030928	0.00706491
Muller M	2	76	0.01754386	0.01640286
Hilaire G	3	101.052673	0.02777778	0.06151613
Bevengut M	3	11.4291055	0.02222222	0.03389591
Dutschmann M	3	90.0625	0.02325581	0.02443086
Viemari JC	3	0	0.01960784	0.02355085
Voituron N	3	12.8642857	0.02272727	0.03456489
Menuet C	3	0.875	0.0212766	0.02687001
Tamirez JM	3	0	0.01960784	0.01497392
Zanella S	3	20.5781417	0.02564103	0.02843496
Burnet H	3	0	0.01960784	0.0205433
Lajard AM	3	5.87440476	0.02173913	0.02252833
Noux JC	3	42	0.02040816	0.01921498
Bou-Flores C	3	0	0.01785714	0.0094492
Gestreau C	3	0.26388889	0.02083333	0.01542891
Monteau R	3	0	0.01785714	0.00788292
del Negro CA	4	0	0.05882353	0.02623163
Kottick A	4	0	0.05882353	0.02362381
Vann NC	4	0	0.05882353	0.02362381
Funk GD	4	20	0.08333333	0.02115704
Hayes JA	4	0	0.05882353	0.02105345
Smith JC	5	14	0.07142857	0.0227787
Richerson GB	5	0	0.04761905	0.01940947
Hodges MR	5	0	0.04761905	0.01518263
Thoby-Brisson M	6	22	0.01923077	0.01934309
Fortin G	6	0	0.01886792	0.01401169
Cardoit L	6	0	0.01351351	0.00671492
Feldman JL	7	8	0.06666667	0.01962096
Janczewski WA	7	0	0.04347826	0.01140013
Onimaru H	8	0	0.2	0.02755422
Arata S	8	0	0.2	0.02279012
Ikeda K	8	0	0.2	0.02245874
Kawakami K	8	0	0.2	0.02245874
Arata A	8	0	0.2	0.01440509
Ogawa M	8	0	0.2	0.01278207
Champagnat J	9	0	0.5	0.02040816
Charnay P	9	0	0.5	0.02040816
Chatonnet F	9	0	0.5	0.02040816
Herlenius E	10	0	0.01428571	0.00887446
Lagercrantz H	10	0	0.01428571	0.00887446

Table S3. Co-citation network results

Reference	Cluster	Betweenness	Closeness	PageRank
viemari jc 2005	1	59.4291197	0.00303951	0.02650208
guy j 2001	1	1.16442619	0.00285714	0.02249876
stettner gm 2007	1	0.39187527	0.00284091	0.02188752
amir re 1999	1	0.61581469	0.00285714	0.01905859
stettner gm 2008	1	17.1741458	0.00295858	0.01206748
katz dm 2009	1	0	0.00282486	0.01644956
smith jc 1991	2	448.18957	0.00326797	0.06055503
gray pa 2001	2	100.454059	0.00309598	0.03224413
gray pa 1999	2	56.1116529	0.0030581	0.04017544
onimaru h 2003	2	37.2616332	0.0030581	0.04003897
feldman jl 2006	2	41.1282279	0.00306748	0.02374581
jacquin td 1996	2	4.81619208	0.00277778	0.03133556
bianchi al 1995	2	2.30306753	0.00268817	0.01352916
dubreuil v 2008	2	9.09452611	0.00289855	0.0294259
thoby-brisson m 2009	2	14.0689876	0.00299401	0.03042304
stornetta rl 2006	2	0.4875	0.00049164	0.02303668
thoby-brisson m 2005	2	1.78257326	0.00284091	0.02207948
amiel j 2003	2	1.21173925	0.00277778	0.02239625
dubreuil v 2009	2	1.8012631	0.0027933	0.01971233
lieske sp 2000	2	3.07066864	0.00289017	0.01699491
rekling jc 1998	2	13.8801214	0.00298507	0.01786255
rose mf 2009	2	2.42909481	0.00283286	0.01877721
viemari jc 2003	2	0.35584795	0.00258398	0.01020836
ballanyi k 1999	2	1.02272345	0.00274725	0.01170771
mellen nm 2003	2	0.33261888	0.00276243	0.02206286
pagliardini s 2008	2	1.72411228	0.00280899	0.02072359
richerson gb 2004	2	2.74977983	0.00273224	0.00983615
suzue t 1984	2	0	0.00045998	0.00872975
viemari jc 2004	2	6.79622857	0.00285714	0.01246471
abbott sbg 2009	2	0	0.00047801	0.01034397
feldman jl 2003	2	0	0.00046816	0.00890864
guyenet pg 2002	2	5.62827642	0.00290698	0.01359269
janczewski wa 2006	2	0.04826415	0.00263158	0.01466267
koshiya n 1999	2	0.84632605	0.0026178	0.00842396
mulkey dk 2004	2	0.1625	0.00048924	0.01511439
pattyn a 1997	2	0.49333333	0.00048924	0.01515194
bou-flores c 2000	3	59.7793609	0.00293255	0.02050189
corcoran ae 2009	3	4.45874655	0.00292398	0.00960694
hilaire g 1999	3	16.3762874	0.00290698	0.01288224
stornetta rl 2003	4	57.6748331	0.00303951	0.01809545
feldman jl 2013	4	188.029494	0.00310559	0.02161332
gray pa 2010	4	104.566452	0.00308642	0.02342499
bouvier j 2010	4	52.7361991	0.00304878	0.02335325
funk gd 1993	4	59.0365334	0.00297619	0.02107879
janczewski wa 2013	4	39.5457209	0.00292398	0.01839515
tan wb 2008	4	4.88345998	0.00289017	0.01950731
greer jj 1991	4	66.3478109	0.00299401	0.01538248
kam k 2013	4	0.95196245	0.00287356	0.01673244
wallen-mackenzie a 2006	4	17.3505926	0.00297619	0.02138071
marchenko v 2016	4	16.2362766	0.00289017	0.01531724

Table S4: Co-occurrence network analysis results

Node	Cluster	Betweenness	Closeness	PageRank
cpg-binding protein-2	1	46.5357143	0.01086957	0.01468734
kolliker-fuse nucleus	1	0	0.01041667	0.0075508
respiratory neurons	2	332.524981	0.01694915	0.10768489
pre-botzinger complex	2	148.045352	0.01538462	0.07964843
mouse	2	59.2959213	0.01265823	0.04007154
in vitro	2	26.732348	0.01265823	0.04338921
respiratory rhythm	2	1.72426813	0.01149425	0.02000463
spinal cord	2	18.0337789	0.01265823	0.03598932
brain	2	5.0816841	0.01136364	0.02193239
modulation	2	2.0567365	0.01176471	0.02280669
rat	2	1.43811734	0.01149425	0.01830825
transgenic mice	2	0.41570089	0.01086957	0.01480121
rett-syndrome	2	0.87059901	0.01123596	0.01414425
mechanisms	2	0.09090909	0.01041667	0.00986211
respiratory network	2	0	0.01020408	0.0060531
generation	2	0.62167636	0.0106383	0.01202252
inhibition	2	0	0.01010101	0.00725043
network	2	0.18249158	0.0106383	0.01039631
neurotrophic factor	2	0	0.01	0.00717434
spinal cord preparations	2	0.37370661	0.01086957	0.01232534
synaptic transmission	2	0	0.01020408	0.00988557
breathing pattern	2	0	0.01	0.00719371
glutamate	2	0	0.00925926	0.00541536
maturation	2	0	0.01010101	0.00715633
brainstem	3	224.795829	0.01612903	0.08392294
respiratory rhythm generation	3	73.2560205	0.01351351	0.05978482
newborn rats	3	19.7787553	0.01265823	0.0398746
serotonin	3	30.6185282	0.01298701	0.0370516
medulla	3	2.31440934	0.01176471	0.02850968
expression	3	4.38263757	0.01162791	0.02038264
in-vivo	3	0.59451587	0.01123596	0.01856126
motoneurons	3	0	0.01086957	0.01034938
infant-death-syndrome	3	0.74164683	0.01098901	0.01338454
nervous-system	3	0.5061083	0.01123596	0.01468576
raphe neurons	3	0.06451613	0.00990099	0.01032419
projections	3	0	0.00952381	0.00602289
substance-p	3	0	0.00934579	0.00551539
phox2b	4	73.7637745	0.0125	0.03673503
retrotrapezoid nucleus	4	0.0625	0.01111111	0.01356202
central hypoventilation syndrome	4	0.09677419	0.01111111	0.01518212
mutations	4	0	0.01020408	0.00693196

expression pattern	4	0	0.00877193	0.00561187
locus-coeruleus	4	0	0.00970874	0.00687298
postnatal-development	5	46	0.01020408	0.01138421
synaptic plasticity	6	0	0.00724638	0.00520571
gaba	7	0	0.00694444	0.00554414
receptors	8	0	0.008	0.00436357
sleep	9	0	0.00793651	0.0044826

Table S5: Potential transgenic lines for the study of respiratory rhythm generation and modulation.

Gene	Phenotype	Genotype ID	Sex	Finding	Zigosity	Expressed in pBc or BS	Refs.	Model of human disease
**Ace2**	Increased pulmonary respiratory rate	MGI:5828264	F	Mice exposed to cigarette smoke for 3 weeks show a greater increase in resting respiratory rate than wild-type mice exposed to smoke.	Homz.	BS	8	NO
**Ada**	Tachypnea	MGI:2683994	USP.	Tachypnea: evident beginning at postnatal day 12 and progressively labored breathing until death.	Com.	BS	10	Adenosine deaminase deficiency
**Adgrf5**	Tachypnea	MGI:5697400	USP.	Tachypnea. Respiratory distress: signs of severe respiratory distress develop between 20 and 32 weeks of age with chest retractions.	Homz.	BS	3	NO
**Agl**	Increased pulmonary respiratory rate	MGI:5661390	USP.	Increased pulmonary respiratory rate: older mice show an accelerated respiratory rate at basal conditions.	Homz.	BS	2	Glycogen storage disease III
**Aldoc**	Increased pulmonary respiratory rate	MGI:6461053	USP.	Increased pulmonary respiratory rate and decreased pulmonary ventilation.	Homz.	BS	3	NO

Continued on next page

Table S5 continued from previous page

Gene	Phenotype	Genotype ID	Sex	Finding	Zigosity	Expressed in pBc or BS	Refs.	Model of human disease
**Arap1**	Decreased pulmonary respiratory rate	MGI:6461071	M	Increased pulmonary respiratory rate and decreased pulmonary ventilation.	Homz.	BS	4	NO
**Arid4a**	Increased pulmonary respiratory rate	MGI:3817454	USP.	Increased pulmonary respiratory rate in mice older than 5 months.	Homz.	BS	3	Acute myeloid leukemia
**Asap1**	Decreased pulmonary respiratory rate	MGI:6358633	USP.	Decreased pulmonary respiratory rate: respiratory rate is significantly reduced at P0. However, respiratory rate is recovered in neonates that survive the first day after birth and surviving pups reach adulthood.	Homz.	BS	4	NO

Continued on next page

Table S5 continued from previous page

Gene	Phenotype	Genotype ID	Sex	Finding	Zigosity	Expressed in pBc or BS	Refs.	Model of human disease
**Atoh1**	Decreased pulmonary respiratory rate	MGI:2183424	USP.	Decreased pulmonary respiratory rate: diaphragmatic electromyograms indicate slow and variable respiratory rhythms involving the phrenic nerve; rhythms also slow when recorded from hypoglossal and C4 nerves when the diaphragm is not part of the experimental preparation.Glutamatergic modulators such as dihydrokainic acid and ampakine (CX546) restore near normal rhythm and pattern in expermimental preparations; substance P increases respiratory rhythm but does not restore normal frequency or pattern; norepinephrin establishes a similar slow, regular pace in both mutant and control experimental preparations. Respiratory failure: animals do not breathe after birth.	Homz.	BS	62	NO
**Bach2**	Increased pulmonary respiratory rate	MGI:6261950	USP.	Increased pulmonary respiratory rate.	Homz.	BS	2	NO

Continued on next page

Table S5 continued from previous page

Gene	Phenotype	Genotype ID	Sex	Finding	Zigosity	Expressed in pBc or BS	Refs.	Model of human disease
**bd**	Decreased pulmonary respiratory rate	MGI:2656184	USP.	Decreased pulmonary respiratory rate: starting at 10 days of age, all bradypneic homozygotes display a ˜50% reduction in breathing frequency relative to heterozygous controls (measured as 130 vs 264 breaths/min, respectively, at 4 weeks of age); polygraph recordings indicate that mutant mice breathe more deeply than control mice; however, sniffing rates are within the normal range; at 3 weeks of age or later, surviving homozygotes breathe at half the normal rate (bradypneic) but breathing is deeper; however, oxygen consumption per unit of body surface, blood pH, and blood CO2 capacity are normal; no obstruction is detected in the nasal passages, larynx, trachea or bronchi.	Homz.	Not a gene	2	NO
**Bdnf**	Decreased pulmonary respiratory rate	MGI:2175721	USP.	Decreased pulmonary respiratory rate: reduced and irregular breathing develops with age.	Homz.	BS	15	NO

Continued on next page

Table S5 continued from previous page

Gene	Phenotype	Genotype ID	Sex	Finding	Zigosity	Expressed in pBc or BS	Refs.	Model of human disease
**Bmp7 and Wnt**	Decreased pulmonary respiratory rate	MGI:6509439	USP.	Abnormal respiration: short respiratory disruptions following a sigh is reduced, whereas prolonged post-sigh events (more than or two apneas following a sigh) are increased. Decreased pulmonary respiratory rate: mice with apneas exhibit a lower baseline respiratory frequency and an increase in cycle duration of each respiratory event due to an increase in the inspiratory time. Apnea: plethysmopgraphy shows that 50% of mice elicit a greater number of spontaneous apneas in normoxia; presence of greater number of spontaneous apneas persists during hypoxia, as well as during the first minute of recovery to normoxia; however, the presence of greater number of spontaneous apneas disappears during hyperoxia.	Con.	BS	16	Obstructive sleep apnea
**Braf**	Tachypnea	MGI:4946647	USP.	Tachypnea at 4 months of age.	Het.	BS	3	Cardiofaciocutaneous syndrome
**Cdc73**	Decreased pulmonary respiratory rate	MGI:3795455	USP.	Decreased pulmonary respiratory rate: after treatment of adults with tamoxifen, breathing is labored.	Con.	BS	5	NO

Continued on next page

Table S5 continued from previous page

Gene	Phenotype	Genotype ID	Sex	Finding	Zigosity	Expressed in pBc or BS	Refs.	Model of human disease
**Chat and Cpeb2**	Decreased pulmonary respiratory rate	MGI:5882505	USP.	Abnormal whole-body plethysmography: whole-body plethysmography revealed respiratory defects similar to those observed in Cpeb2tm1.2Yshu homozygotes but with wide individual variation in severity; however, tidal volume is normal at P1. Abnormal breathing pattern: aberrant respiration patterns at P1.Decreased pulmonary respiratory rate: reduced respiratory frequency at P1. Apnea: increased apneic episodes at P1; inhalation of nebulized tiotropium, an anticholinergic bronchodilator, reduces apnea frequency to wild-type levels.	Con.	BS	2	NO
**Chd2**	Tachypnea	MGI:3838399	USP.	Tachypnea as early as 16 weeks of age for lung edema.	Het.	BS	5	NO
**Cmtm6**	Abnormal pulmonary respiratory rate	MGI:6511251	USP.	Abnormal pulmonary respiratory rate: absence of spontaneous apnea.	Con.	BS	1	NO

Continued on next page

Table S5 continued from previous page

Gene	Phenotype	Genotype ID	Sex	Finding	Zigosity	Expressed in pBc or BS	Refs.	Model of human disease
**Cpeb2**	Decreased pulmonary respiratory rate	MGI:5882503	USP.	Abnormal whole-body plethysmography: whole-body plethysmography revealed severe respiratory defects, similar to those observed in Cpeb2tm1.2Yshu homozygotes; however, tidal volume is normal at P1 Abnormal breathing pattern: pups exhibit aberrant respiration patterns at P1. Decreased pulmonary respiratory rate: significantly reduced respiratory frequency at P1. Apnea: significantly increased apneic episodes at P1. Respiratory failure	Con.	BS	3	NO

Continued on next page

Table S5 continued from previous page

Gene	Phenotype	Genotype ID	Sex	Finding	Zigosity	Expressed in pBc or BS	Refs.	Model of human disease
**Cpeb2**	Decreased pulmonary respiratory rate	MGI:5882502	USP.	At postnatal day 1 (P1), pups exhibit abnormal breathing patterns characterized by prolonged inspiratory and expiratory times, reduced peak inspiratory and expiratory flow, and decreased respiratory frequency. These abnormalities are observed under both normoxic and hypercapnic conditions. However, lung morphology remains normal with properly inflated alveoli. The pups also experience increased apneic episodes and duration at P1, which can be partially rescued by the inhalation of nebulized tiotropium, an anticholinergic bronchodilator. The nervous system appears largely unaffected, with normal morphology of the pBc and parafacial respiratory group (pFRG) and stable rhythmic activities in C4 ventral roots. There are no significant alterations in pontine and medullary (nor)adrenergic neurons or diaphragm neuromuscular junctions. These findings suggest that the observed respiratory abnormalities are primarily related to lung and breathing control mechanisms rather than nervous system dysfunction.	Homz.			NO

Continued on next page

Table S5 continued from previous page

Gene	Phenotype	Genotype ID	Sex	Finding	Zigosity	Expressed in pBc or BS	Refs.	Model of human disease
**Csnk1g2**	Increased pulmonary respiratory rate	MGI:6262217	USP.	Increased pulmonary respiratory rate.	Homz.	BS	2	NO
**Decr2**	Increased pulmonary respiratory rate	MGI:6461201	M	Increased pulmonary respiratory rate.	Homz.	BS	2	NO
**Dhcr7**	Decreased pulmonary respiratory rate	MGI:3774001	USP.	Abnormal breathing pattern: breathing is irregular and shallow. Decreased pulmonary respiratory rate: breathing frequency is slower Apnea: breathing is irregular and shallow, with long periods of apnea. Abnormal lung morphology.	Homz.	BS	18	NO
**Dnm1l**	Tachypnea	MGI:4818928	USP.	Pleural effusion: pleural effusion in some CHF mutant mice. Tachypnea: shallow rapid breathing.	Het.	BS	2	Dilated cardiomyopathy
**Drd1**	Decreased pulmonary respiratory rate	MGI:3709752	USP.	Decreased pulmonary respiratory rate: at P2 mice exhibit periodic breathing that is resolved in older pups.	Con.	BS		NO

Continued on next page

Table S5 continued from previous page

Gene	Phenotype	Genotype ID	Sex	Finding	Zigosity	Expressed in pBc or BS	Refs.	Model of human disease
**Drd2**	Increased pulmonary respiratory rate	MGI:4429667	USP.	Increased tidal volume: under hypercapnic conditions in male, but not female, mice: under hypoxic hypercapnic conditions in female, but not male mice. Increased pulmonary respiratory rate: under hypoxic hypercapnic conditions in female, but not male mice. Decreased pulmonary ventilation: in male mice at day 2 and 8 of exposure to poikilocapnic hypoxia. Increased pulmonary ventilation: at low levels of hypoxia in male mice; under hypoxic conditions in female, but not male mice; under hypercapnic conditions in male, but not female, mice; under hypoxic hypercapnic conditions in female, but not male mice.	Homz.	BS	101	Parkinson's disease
**Dscam**	Decreased pulmonary respiratory rate	MGI:3840675	USP.	Apnea: at birth, mice exhibit frequent apenic episodes unlike wild-type mice. Respiratory distress: at birth, mice exhibit irregular respiratory rhythm and frequent apneic episodes unlike wild-type mice. Background Sensitivity: mice exhibit irregular breathing patterns that are more severe than in mice on a mixed 129P2/OlaHsd, BALB/c, and C57BL/6 background.	Homz.	BS	5	NO

Continued on next page

Table S5 continued from previous page

Gene	Phenotype	Genotype ID	Sex	Finding	Zigosity	Expressed in pBc or BS	Refs.	Model of human disease
**Eef2**	Increased pulmonary respiratory rate	MGI:6771973	USP.	Increased pulmonary respiratory rate.	Homz.	BS	3	NO
**Egr2**	Increased pulmonary respiratory rate	MGI:3803096	USP.	Increased pulmonary respiratory rate: mice exhibit high-frequency breathing at around 3 weeks of age that leads to death.	Homz.	BS	1	NO
**Epas1**	Increased pulmonary respiratory rate	MGI:5518458	USP.	Increased pulmonary respiratory rate: under hypoxic, but not normoxic, conditions.	Homz.	BS	1	NO
**Epc2**	Increased pulmonary respiratory rate	MGI:5757053	USP.	Increased pulmonary respiratory rate	Het.	BS	3	NO

Continued on next page

Table S5 continued from previous page

Gene	Phenotype	Genotype ID	Sex	Finding	Zigosity	Expressed in pBc or BS	Refs.	Model of human disease
**Erbb4**	Decreased pulmonary respiratory rate	MGI:3690032	USP.	Abnormal respiratory function: at 11-14 weeks of age, tidal midexpiratory flow is significantly reduced relative to that in heterozygous controls. Decreased pulmonary respiratory rate: at 11-14 weeks of age, respiratory frequency (breaths/min) is significantly reduced relative to that in heterozygous controls.	Com.	BS	49	NO
**Flnb**	Tachypnea	MGI:5660810	USP.	tachypnea: mice exhibit tachypnea, likely as a result of thoracic deformities.	Homz.	BS	1	NO
**Foxd1**	Tachypnea	MGI:3037870	USP.	Tachypnea: several hours after birth, homozygotes develop a fast respiratory rate.	Homz.	BS	13	NO
**Foxp2**	Increased pulmonary respiratory rate	MGI:3851148	USP.	Increased pulmonary respiratory rate: mice exhibit higher respiratory rate during activity and at rest compared with wild-type mice.	Het.	BS	3	NO
**Glra1**	Abnormal pulmonary respiratory rate	MGI:3810131	USP.	Abnormal pulmonary respiratory rate: conscious mutants (P16-23) display disturbed respiratory patterns characterized by periods of slow breathing and intermittent episodes of rapid breathing; episodes are of variable (2-10 sec).	Homz.	BS	17	NO

Continued on next page

Table S5 continued from previous page

Gene	Phenotype	Genotype ID	Sex	Finding	Zigosity	Expressed in pBc or BS	Refs.	Model of human disease
**Gna11/Gnaq**	Decreased pulmonary respiratory rate	MGI:3037555 MGI:3037556	USP. USP.	Abnormal breathing pattern: arrhythmic breathing. Decreased pulmonary respiratory rate: animals breathed at approximately half the rate of controls.	Com. Com.	BS	65	NO
**Grin1**	Abnormal pulmonary respiratory rate	MGI:3845918	USP.	Abnormal breathing pattern. Abnormal pulmonary respirator rate. Respiratory distress.	Het.	BS	2	NO
**Hcrt**	Increased pulmonary respiratory rate	MGI:3763455	USP.	Increased pulmonary respiratory rate: respiratory frequency is significantly increased.	Homz.	BS	70	Narcolepsy
**Hdac1 and Hdac2**	Increased pulmonary respiratory rate	MGI:5485404 MGI:5485403	USP.USP.	Increased pulmonary respiratory rate: in moribund mice.	Con. Con.	BS	3	NO
**Hif1an**	Increased pulmonary respiratory rate	MGI:4459903	USP.	Abnormal lung volume: minute volume (Vi) shows that the greatest discrepancy between wild-type and mutant mice at normoxia. Increased tidal volume: at normoxia. Increased pulmonary respiratory rate: elevation in frequency of respiration only during hypoxia.	Homz.	BS	4	NO

Continued on next page

Table S5 continued from previous page

Gene	Phenotype	Genotype ID	Sex	Finding	Zigosity	Expressed in pBc or BS	Refs.	Model of human disease
**Hmox2**	Decreased pulmonary respiratory rate	MGI:3612981	USP.	Abnormal respiration: lung architecture and ventilation normal; less responsive to acute hypoxia. Abnormal vital capacity: lower tidal volume and minute ventilation. Decreased pulmonary respiratory rate: lower respiratory frequency.	Homz.	BS	52	NO

Continued on next page

Table S5 continued from previous page

Gene	Phenotype	Genotype ID	Sex	Finding	Zigosity	Expressed in pBc or BS	Refs.	Model of human disease
**Ighmbp2**	Decreased pulmonary respiratory rate	MGI:7314416	USP.	Increased tidal volume: under normoxia conditions, mice show higher tidal volume that is increased further under hypercapnia + hypoxia conditions. Abnormal respiratory function: mice challenged with hypercapnia + hypoxia, do not respond by increasing ventilation and mean inspiratory flow as seen in wild-type mice. Decreased pulmonary respiratory rate: under normoxia conditions, mice have decreased respiratory frequency; respiratory frequency decreases further when challenged with hypercapnia + hypoxia conditions instead of increasing as in wild-type mice. Apnea: number of apneas is increased under normoxia conditions but not during hypercapnia + hypoxia; ICV injection of the high dose of ssAAV9-IGHMBP2 improves respiratory frequency, the number of apneas and erratic breathing nearly to wild-type. Decreased pulmonary ventilation: mice challenged with hypercapnia + hypoxia, do not respond by increasing ventilation as in wild-type mice.	Homz.	BS	1	Distal spinal muscular atrophy 1

Continued on next page

Table S5 continued from previous page

Gene	Phenotype	Genotype ID	Sex	Finding	Zigosity	Expressed in pBc or BS	Refs.	Model of human disease
	Decreased pulmonary respiratory rate	MGI:3603515	USP.	Decreased pulmonary respiratory rate: reduced breathing rate (bradypnea).	Homz.		11	Distal spinal muscular atrophy 1
	Decreased pulmonary respiratory rate	MGI:3765069	USP.	Decreased pulmonary respiratory rate: reduced breathing rate (bradypnea).	Com.			NO
	Decreased pulmonary respiratory rate	MGI:3785251	USP.	Decreased pulmonary respiratory rate.	Com.			NO
**Ighmbp2 and Mnm**	Decreased pulmonary respiratory rate	MGI:3603516	USP.	Pulmonary edema: consolidation of lungs. Decreased pulmonary respiratory rate: reduced breathing rate (bradypnea).	Com.			NO
**Ilf3**	Tachypnea	MGI:3604750	USP.	Tachypnea: breathing is rapid and shallow; however lung development and lung inflation are normal.	Homz.	BS	3	NO
**Jak2**	Tachypnea	MGI:6356966	USP.	Tachypnea: in moribund mice.	Con.	BS	467	Myeloproliferative neoplasm

Continued on next page

Table S5 continued from previous page

Gene	Phenotype	Genotype ID	Sex	Finding	Zigosity	Expressed in pBc or BS	Refs.	Model of human disease
**Kcnj3**	Decreased pulmonary respiratory rate	MGI:6461367	M	Decreased pulmonary ventilation.	Homz.	BS	3	NO
**Kcnn3**	Abnormal pulmonary respiratory rate response	MGI:3524861	USP.	Abnormal pulmonary respiratory rate response: without doxycycline treatment homozygotes are unable to sustain a hypoxia-induced increase in breathing frequency; after doxycycline treatment for 6 days response to hypoxia is normal. Apnea: without doxycycline treatment 3 out of 7 homozygotes had apneic episodes when exposed to hypoxic conditions.	Homz.	BS	19	NO
**Klf5**	Increased pulmonary respiratory rate	MGI:4421819	USP.	Increased pulmonary respiratory rate: following high-intensity transverse aortic constriction compared to in similarly treated Klf5tm2.1Rng homozygotes.	Con.	BS	14	NO
**Kras, Pten, and Scgb1a1**	Tachypnea	MGI:4839216	USP.	Tachypnea: by 2 months of age, mutants exhibit tachypnea.	Con.	BS	25	NO

Continued on next page

Table S5 continued from previous page

Gene	Phenotype	Genotype ID	Sex	Finding	Zigosity	Expressed in pBc or BS	Refs.	Model of human disease
**Lama2**	Tachypnea	MGI:6226150	USP.	Tachypnea: mice present polypnea in the last 3 weeks of life.	Homz.	BS	1	Congenital merosin-deficient muscular dystrophy 1A
**Lmna**	Tachypnea	MGI:3527796	Both	Tachypnea: males and females began to exhibit rapid shallow breathing at 3 months and 6 months of age, respectively.	Homz.	BS	32	Dilated cardiomyopathy 1A and emery-Dreifuss muscular dystrophy
**Mcrip1**	Tachypnea	MGI:6358238	USP.	Tachypnea: most newborn pups exhibit tachypnea. Respiratory distress: most newborn pups exhibit gasping breath. Respiratory failure: most homozygotes die at the neonatal stage due to respiratory failure.	Homz.	BS	2	NO
**Mecp2**	Increased pulmonary respiratory rate	MGI:6472629	M	Abnormal breathing pattern: mice show increased breathing irregularity. Increased pulmonary respiratory rate: mice show increased basal breathing rate. Apnea: mice show elevated incidence of apneas.	Other (hemizygous, indeterminate, etc.)	BS	1	Rett syndrome

Continued on next page

Table S5 continued from previous page

Gene	Phenotype	Genotype ID	Sex	Finding	Zigosity	Expressed in pBc or BS	Refs.	Model of human disease
	Increased pulmonary respiratory rate	MGI:5499849	USP.	Increased pulmonary respiratory rate: even in mice treated with fluvastatin.	Other (hemizygous, indeterminate, etc.)		272	NO
	Increased pulmonary respiratory rate	MGI:5499839	USP.	Increased pulmonary respiratory rate: as in Mecp2tm1.1Bird hemizygotes.	Com.			NO
	Increased pulmonary respiratory rate	MGI:5751707	M	Increased pulmonary respiratory rate: 6-8 week old mice have increased breathing rate at baseline and when exposed to a hypoxic gas challenge.	Other (hemizygous, indeterminate, etc.)		4	Rett syndrome
	Increased pulmonary respiratory rate	MGI:5751716	F	Increased pulmonary respiratory rate: 8 month old mice exhibit increased breathing rate when exposed to a hypoxic gas challenge.	Het.			Rett syndrome

Continued on next page

Table S5 continued from previous page

Gene	Phenotype	Genotype ID	Sex	Finding	Zigosity	Expressed in pBc or BS	Refs.	Model of human disease
	Tachypnea	MGI:3624676	F	Abnormal breathing pattern: mutants at 10 weeks of age exhibit an abnormally high breathing frequency associated with marked decreases in expiratory time and total breath duration and a small but significant decrease in inspiratory time; treatment with LM22A-4, a small molecule BDNF loop domain mimetic that acts as a selective TrkB agonist, improves the respiratory function of mutants. Tachypnea: breathing dysfunction is characterized by increased frequency due to periods of tachypnea and increased apneas. Apnea: 20% of mutants exhibit apneas at 8 weeks of age and by 12 weeks of age, 50% of mutants exhibit apneas; number of apneas increases between 8 and 12 weeks of age in mutants.	Het.		92	Rett syndrome
**Mir34a**	Decreased pulmonary respiratory rate	MGI:5791913	F	Fs show slightly lower respiratory rates, slightly lower tidal volumes and minute ventilation volumes at rest. However, mice show normal adaptation to activity. Fs show slightly lower minute ventilation volumes at rest.	Homz.	BS	1	NO

Continued on next page

Table S5 continued from previous page

Gene	Phenotype	Genotype ID	Sex	Finding	Zigosity	Expressed in pBc or BS	Refs.	Model of human disease
**Mpig6b**	Increased pulmonary respiratory rate	MGI:6461443	M	Increased pulmonary respiratory rate.	Homz.	BS	3	NO
**Ndufs4**	Decreased pulmonary respiratory rate and Abnormal pBc physiology	MGI:5451011<br>MGI:5451025	USP.	Mutants exhibit intermittent breathing irregularities, including hypo- or hyperventilation, variable and lower breathing rates, increased apnea episodes with age, and gasping-like episodes under stress. Their responses to hypoxic conditions show a decreased amplitude of fictive gasping and abnormal intracellular recordings of inspiratory neurons in the preBotzinger complex. Compared to controls, mutants have reduced depolarization, lower action potentials per burst, and firing frequency under normal conditions. When challenged by hypoxia, mutants experience a severe reduction in depolarization, leading to a significant drop in action potentials per burst, whereas controls exhibit only a mild reduction. These findings suggest impaired respiratory control mechanisms in mutants.	Homz.<br>Con.	pBc	45	Leigh disease

Continued on next page

Table S5 continued from previous page

Gene	Phenotype	Genotype ID	Sex	Finding	Zigosity	Expressed in pBc or BS	Refs.	Model of human disease
**Nos1**	Abnormal pulmonary respiratory rate response and Increased pulmonary respiratory rate	MGI:2174975	USP.	Anesthetized mice under 21% or 12% oxygen show period increases in respiratory rate not seen in wild-type mice. In awake mice, the magnitude of the decrease in respiratory rate following brief exposure to 100% oxygen was greater than that in wild-type mice. In awake and anesthetized mice the magnitude of the increases in respiratory rate at 21% and 12% oxygen are greater than in wild-type controls. Anesthetized mice do not display respiratory depression under 12% oxygen, unlike wild-type controls. In anesthetized mutant mice the increase in ventilation under 21% oxygen results from an increase in both respiratory rate and tidal phrenic activity, unlike in wild-type mice where only tidal phrenic activity is increased. Sodium cyanide induced respiratory stimulation is increased in mutants compared to wild-type mice.	Homz.	BS	287	Hypertrophic pyloric stenosis
**Nrxn1**	Decreased pulmonary respiratory rate	MGI:5757433	USP.	Decreased pulmonary respiratory rate.	Homz.	pBc	3	NO

Continued on next page

Table S5 continued from previous page

Gene	Phenotype	Genotype ID	Sex	Finding	Zigosity	Expressed in pBc or BS	Refs.	Model of human disease
**Nucks1**	Increased pulmonary respiratory rate	MGI:6295177	USP.	Increased pulmonary respiratory rate.	Homz.	BS	1	NO
**Or51e2**	Abnormal pulmonary respiratory rate	MGI:5903955	USP.	Abnormal pulmonary respiratory rate.	Homz.	BS	6	NO
**Parp12**	Increased pulmonary respiratory rate	MGI:6461512	M	Increased pulmonary respiratory rate.	Homz.	BS	3	NO
**Pfn4**	Increased pulmonary respiratory rate	MGI:6461524	F	Increased pulmonary respiratory rate.	Homz.	BS	3	NO
**Phox2a**	Decreased pulmonary respiratory rate	MGI:3845071	USP.	Duration of the respiratory cycle is more irregular for E18 preterm neonates. Mean respiratory frequency is reduced for E18 preterm neonates.	Homz.	BS	4	NO

Continued on next page

Table S5 continued from previous page

Gene	Phenotype	Genotype ID	Sex	Finding	Zigosity	Expressed in pBc or BS	Refs.	Model of human disease
	Increased pulmonary respiratory rate	MGI:3845087	USP.	Breathing frequency shows a greater initial increase under hypoxic conditions than controls.	Het.			NO
**Phox2b**	Decreased pulmonary respiratory rate	MGI:7397265	USP.	Mice show spontaneous/continuous breathing, albeit at a slightly reduced frequency, and do not exhibit cyanosis. BSs show loss of the retrotrapezoid nucleus.	Con.	BS	4	NO
**Phpt1**	Increased pulmonary respiratory rate	MGI:6461526	USP.	Increased pulmonary respiratory rate.	Homz.	BS	3	NO
**Pitx3**	Increased pulmonary respiratory rate	MGI:4429423	M	M mice exhibit increased mean respiratory rates and decreased mean inspiratory flow, minute ventilation, inspiratory time, and tidal volume compared with wild-type mice. M mice exhibit increased mean respiratory rates and decreased mean inspiratory flow, minute ventilation, inspiratory time, and tidal volume compared with wild-type mice. Mice exhibit more frequent but less effective breathing compared with wild-type mice.	Homz.	BS	1	Parkinson's disease

Continued on next page

Table S5 continued from previous page

Gene	Phenotype	Genotype ID	Sex	Finding	Zigosity	Expressed in pBc or BS	Refs.	Model of human disease
**Pnck**	Decreased pulmonary respiratory rate	MGI:6480552	USP.	Decreased pulmonary respiratory rate.	Other (hemizygous, indeterminate, etc.)	BS	3	NO
**Pwwp2b**	Decreased pulmonary respiratory rate	MGI:6867797	USP.	Decreased pulmonary respiratory rate: during the day and night in mice fed a high-fat diet compared with control mice.	Con.	BS	1	NO
**Rpl13a and Lyz2**	Tachypnea	MGI:5502329	USP.	Increased breathing rate.	Con.	BS	3	NO
**Rxfp2**	Increased pulmonary respiratory rate	MGI:5757671	USP.	Increased pulmonary respiratory rate.	Homz.	BS	3	NO
**Ryr1**	Increased pulmonary respiratory rate	MGI:4887395	USP.	Increased pulmonary respiratory rate: resulting from halothane anesthesia; rate increase from 147 to about 183 breaths per minute.	Het.	BS	8	malignant hyperthermia

Continued on next page

Table S5 continued from previous page

Gene	Phenotype	Genotype ID	Sex	Finding	Zigosity	Expressed in pBc or BS	Refs.	Model of human disease
**Scube3**	Decreased pulmonary respiratory rate	MGI:5817790	F	Decreased pulmonary respiratory rate. Decrease in respiration rate in females.	Homz.	BS	3	NO
**Sepsecs**	Decreased pulmonary respiratory rate	MGI:7367146	USP.	Decreased pulmonary respiratory rate. Shallow and irregular breathing develops after birth. However, mice are born with normal breathing frequency.	Homz.	BS	1	NO
**Sgca**	Decreased pulmonary respiratory rate	MGI:7278768	USP.	Decreased pulmonary respiratory rate; mice show lower respiration rates at 15 and 34 weeks of age; respiration rate is lower than in Sgcdtm1Mcn homozygotes.	Homz.	BS	3	Autosomal recessive limb-girdle muscular dystrophy type 2D

Continued on next page

Table S5 continued from previous page

Gene	Phenotype	Genotype ID	Sex	Finding	Zigosity	Expressed in pBc or BS	Refs.	Model of human disease
**Sgcd**	Decreased pulmonary respiratory rate	MGI:5911876	USP.	Decreased pulmonary respiratory rate; mice show lower respiration rates at 15 and 34 weeks of age; however, respiration rate is higher than in Sgcatm2Kcam homozygotes.	Homz.	BS	39	Autosomal recessive limb-girdle muscular dystrophy type 2F and dilated cardiomyopathy 1L

Continued on next page

Table S5 continued from previous page

Gene	Phenotype	Genotype ID	Sex	Finding	Zigosity	Expressed in pBc or BS	Refs.	Model of human disease
**Slc6a9**	Decreased pulmonary respiratory rate	MGI:2682534	USP.	Newborn homozygotes display a severe breathing irregularity. However, no histologic abnormalities of the airways or lungs are observed and the musculo-skeletal system appears unaffected. Newborn homozygotes show a severe depression of respiratory frequencies to only 16% of those in wild-type pups, as shown by whole-body plethysmography. Although durations of single breaths are only marginally longer, expiratory intervals are significantly prolonged as shown by a 4-fold increase of the coefficient of variation relative to that in control littermates. Newborn homozygotes exhibit long periods of apnea interrupted by gasp-like inspirations. Newborn homozygotes exhibit long periods of apnea interrupted by gasp-like inspirations.	Homz.	BS	1	Glycine encephalopathy
**Sox11**	Decreased pulmonary respiratory rate	MGI:3053114	USP.	Decreased pulmonary respiratory rate. Only sporadic breathing is seen.	Homz.	BS	11	NO

Continued on next page

Table S5 continued from previous page

Gene	Phenotype	Genotype ID	Sex	Finding	Zigosity	Expressed in pBc or BS	Refs.	Model of human disease
**Syt17**	Increased pulmonary respiratory rate	MGI:6461722	F	Increased pulmonary respiratory rate.	Homz.	BS	4	NO
**Taar6**	Increased pulmonary respiratory rate	MGI:6461724	M	Increased pulmonary respiratory rate.	Homz.	Not reported	4	NO
**Tacr1**	Abnormal pulmonary respiratory rate response	MGI:3619726	USP.	Abnormal pulmonary respiratory rate response. Respiratory frequency fails to increase in adult mice in response to hypoxia.	Homz.	BS	43	NO
**Chrne**	Increased pulmonary respiratory rate	MGI:5478770	USP.	Mutants exhibit a faster respiratory rate by 6-8 weeks of age.	Involves transgenes	BS	8	Congenital myasthenic syndrome 4A

Continued on next page

Table S5 continued from previous page

Gene	Phenotype	Genotype ID	Sex	Finding	Zigosity	Expressed in pBc or BS	Refs.	Model of human disease
**DMWD, DMPK, and SIX5 (DMAHP)**	Decreased pulmonary respiratory rate	MGI:5523468	USP.	Mutants carrying more than 1300 CTG repeats exhibit decreased respiratory rate under anesthesia. Abnormal phrenic nerve morphology: severe loss (41%) of phrenic nerve unmyelinated axons in mutants carrying more than 1300 CTG repeats; phrenic nerves of mutants carrying more than 1300 CTG repeats exhibit aberrant Schwann cell proliferation and increased number of macrophages, and reduced myelin sheath thickness; however, phrenic motor and brainstem respiratory neurons appear normal.	Involves transgenes	BS	20	Myotonic dystrophy type 1
**Fosl2**	Tachypnea	MGI:3813506	USP.	Tachypnea; at 17 weeks mice exhibit signs of respiratory distress such as tachypnea and hunched posture. Abnormal lung morphology.	Involves transgenes	BS	11	Idiopathic pulmonary fibrosis
**GCSAM**	Increased pulmonary respiratory rate	MGI:5642052	USP.	Increased pulmonary respiratory rate: in ill mice.	Involves transgenes	BS	1	Amyloidosis
**Gnaq**	Tachypnea	MGI:2654385	USP.	Tachypnea: 3 of 6 double mutants become tachypneic between 11 and 14 weeks of age. Respiratory distress: 3 of 6 double mutants develop severe respiratory distress between 11 and 14 weeks of age.	Com.	BS	17	NO

Continued on next page

Table S5 continued from previous page

**Gene**	**Phenotype**	**Genotype ID**	**Sex**	**Finding**	**Zigosity**	**Expressed in pBc or BS**	**Refs.**	**Model of human disease**
**Tnf**	Tachypnea	MGI:3586618	USP.	Tachypnea: increased respiration.	Involves transgenes	BS	4	Congestive heart failure
	Tachypnea	MGI:3586619	USP.	Tachypnea: increased respiration.	Involves transgenes		2	Congestive heart failure
**Scgb1a1 and EGFR**	Tachypnea	MGI:5705253	USP.	Mice on a doxycycline (dox)-containing diet for 17.5 weeks become tachypneic.	Com.	Not reported	4	Lung cancer
**Tgfb1**	Decreased pulmonary respiratory rate	MGI:2174884	USP.	Decreased pulmonary respiratory rate.	Involves transgenes	BS	2	NO
**Thpo**	Increased pulmonary respiratory rate	MGI:6461740	USP.	Increased pulmonary respiratory rate.	Homz.	BS	3	NO

Continued on next page

Table S5 continued from previous page

Gene	Phenotype	Genotype ID	Sex	Finding	Zygosity	Expressed in pBc or BS	Refs.	Model of human disease
**Tlx3**	Tachypnea	MGI:2669096		C4 vetral root recordings in medulla-spinal cord preparations from newborn homozygotes reveal a rapid respiratory rate with shorter inspiratory duration and intermittent respiratory arrest of ~7.4 sec in duration relative to wild-type controls. Tachypnea: during non-apnea periods, homozygotes display an increased respiratory rate relative to wild-type mice. Apnea: most newborn homozygotes display immediate apnea; electromyographic activity of intercostal muscles indicates a high incidence of apnea episodes of up to 20 sec in duration; mutant pups exhibit an average of ~13 apnea episodes of more than or equal to 5 sec during 10 min of observation relative to only 1 episode in wild-type mice; the average duration of an apnea episode is nearly doubled in mutant mice relative to wild-type mice. Abnormal nervous system physiology: newborn homozygotes display a functional disorder in the central pattern generator of respiration in the ventral medulla; a coordinate pattern is observed in which failure of inspiratory neuron firing correlates with the respiratory arrest measured as C4 ventral root activity.	Homz.	BS	14	Congenital central hypoventilation syndrome

Continued on next page

Table S5 continued from previous page

Gene	Phenotype	Genotype ID	Sex	Finding	Zigosity	Expressed in pBc or BS	Refs.	Model of human disease
**Tph2**	Decreased pulmonary respiratory rate	MGI:3849845	USP.	Decreased pulmonary respiratory rate: between 12 a.m. and 6 p.m.	Homz.	BS	9	NO
	Decreased pulmonary respiratory rate	MGI:3849846	USP.	Decreased pulmonary respiratory rate: respiration rate (breaths per minute) is decreased compared to in wild-type mice; however, the circadian rhythm of respiration rates is normal. Autonomic control of sleep, breathing, thermoregulation, heart rate, and blood pressure is impaired.	Homz.			NO
**Tpp1**	Tachypnea	MGI:3606473	F	Tachypnea: at 121-149 days of age, mainly homozygous females. Respiratory distress: gasping at 100-130 days of age, homozygous female.	Homz.	BS	1	NO
**Unc13a**	Decreased pulmonary respiratory rate	MGI:2654103	USP.	Neonatal animals exhibit reduced breathing rate.	Homz.	BS	34	NO
**Zfp36 and Lyz2**	Tachypnea	MGI:5441587	USP.	Tachypnea: dramatic in LPS-treated mice.	Con.	BS	9	NO

Continued on next page

Table S5 continued from previous page

Gene	Phenotype	Genotype ID	Sex	Finding	Zigosity	Expressed in pBc or BS	Refs.	Model of human disease
**Zscan10**	Decreased pulmonary respiratory rate	MGI:5707686	M	Decreased pulmonary respiratory rate: in male mice.	Homz.	BS	1	NO
**Cplx1-3**	Abnormal pBc physiology	MGI:2679648	USP.	The amplitude of spontaneous excitatory postsynaptic currents are decreased in pBc neurons compared to in wild-type mice. Tthe amplitude of spontaneous excitatory postsynaptic currents are decreased in pBc neurons compared to in wild-type mice. Spontaneous miniature inhibitory postsynaptic current frequencies are decreased in hippocampal and pBc neurons compared to in wild-type mice.	Com.	pBc	18, 16, 11	NO
	Abnormal pBc physiology	MGI:3807187	USP.	The amplitude and frequency of spontaneous excitatory postsynaptic currents are decreased in pBc neurons compared to in wild-type mice. Spontaneous mEPSC frequencies are decreased in hippocampal neurons and pBc neurons compared to in wild-type.	Com.			NO

Continued on next page

Table S5 continued from previous page

Gene	Phenotype	Genotype ID	Sex	Finding	Zigosity	Expressed in pBc or BS	Refs.	Model of human disease
**Dbx1**	Abnormal pBc physiology and Abnormal pBc morphology	MGI:4837352	USP.	The pBc exhibit abnormal differentiation compared to in wild-type mice. Mice exhibit impaired pBc commissural connectivity detected by biocytin staining compared with wild-type mice. Mice exhibit a reduction in glutamatergic interneurons in the pBc compared with wild-type mice. Mice exhibit impaired pBc commissural connectivity detected by biocytin staining compared with wild-type mice. Mice exhibit a reduction in glutamatergic interneurons in the pBc compared with wild-type mice. At E15.5, mice fail to exhibit fetal breathing (measured by electrophysiological recordings and calcium imaging of the pBc) unlike wild-type mice.	Homz.	pBc	34	NO
	Abnormal pBc physiology and Abnormal pBc morphology	MGI:4837353	USP.	Mice exhibit reduced biocytin stained commissural interneurons in the pBc compared with wild-type mice. Mice exhibit reduced biocytin stained commissural interneurons in the pBc compared with wild-type mice. Mice exhibit de-synchronization of rhythmic activity in the left and right pBc compared with wild-type mice.	Con.			NO

Continued on next page

Table S5 continued from previous page

Gene	Phenotype	Genotype ID	Sex	Finding	Zigosity	Expressed in pBc or BS	Refs.	Model of human disease
**Kdm6b**	Abnormal pBc physiology, Abnormal pBc morphology, and Abnormal nervous system physiology	MGI:5491455	USP.	Disrupted formation and maintenance of the pBc. The respiratory rhythm generator fails to produce rhythmic phrenic bursts unlike in wild-type mice. Electrical shock fails to stimulate phrenic motor neurons unlike in wild-type mice. Medullary stimulation fails to induce phrenic bursts unlike in wild-type mice. Application of acidified artificial cerebrospinal fluid with or without serotonin or norepinephrine or elevated concentrations of potassium fails to induce rhythmic phrenic burst unlike in wild-type mice. However, medullary stimulation commonly induced brief responses of phrenic motor neurons.	Homz.	pBc	3	NO
**Ndn**	Abnormal pBc physiology and Abnormal central pattern generator function	MGI:2653055	USP.	Pups with abnormal breathing fail to generate rhythmic motor bursts from cervical or hypoglossal nerve roots in vitro or generate a severely irregular rhythmic motor output. The respiratory motor discharge pattern produced by cervical ventral roots, hypoglossal roots, cranial motoneuron pools and within neurons located in the putative respiratory rhythm-generating center, the pBc, is very irregular in mice inheriting the mutant allele from the father, with prominent bouts of depression of respiratory rhythmogenesis.	Het.	pBc	11	Prader-Willi syndrome

Continued on next page

Table S5 continued from previous page

Gene	Phenotype	Genotype ID	Sex	Finding	Zigosity	Expressed in pBc or BS	Refs.	Model of human disease
**Nlg1-3**	Abnormal pBc physiology	MGI:3688634	M	Mice show irregular and flat breathing movements. Ratio of glutamatergic vs GABAergic/glycinergic terminals is reduced in the PBC and inferior olive in mutants, but not in hypoglossal neurons. There is a 30% reduction in number of postsynaptic clusters containing GABAAR alpha 1 in the PBC. spontaneous postsynaptic currents from pBc (PBC) neurons are reduced in frequency and duration compared to single knockout animals.Number of GABAergic and glycinergic sPSCs with lower and higher amplitudes are decreased by 85% and 95% respectively. Frequency of GABAergic and glycinergic miniature PSCs (mPSCs) in PBC neurons is reduced by 80% compared to single knockout controls cells. Frequency of spontaneous glutamatergic postsynaptic currents is decreased compared to single null PBC neurons. Amplitudes of GABAergic/glycinergic evoked postsynaptic currents (ePSCs) are strongly decreased compared to single knockout neurons, while amplitudes in double knockout neurons is not significantly changed.	Com.	pBc	26, 25, 8	NO

Continued on next page

Table S5 continued from previous page

**Gene**	**Phenotype**	**Genotype ID**	**Sex**	**Finding**	**Zigosity**	**Expressed in pBc or BS**	**Refs.**	**Model of human disease**
**Robo3**	Abnormal pBc physiology and Abnormal breathing pattern	MGI:4837351	USP.	At P0, mice exhibit independent left and right rhythmic contractions of the diaphragm unlike wild-type mice. At E15.5, rhythmic activity frequency in the pBc is lower than in wild-type mice. At E15.5, bilateral synchronization of rhythmic activity in the pBc is absent unlike in wild-type mice.	Homz.	pBc	23	NO
**Slc17a6**	Abnormal pBc physiology and Respiratory failure	MGI:3694901	USP.	Fail to begin breathing. Absence of spontaneous electrical activity in the hypoglossal root nerve and accompanying changes in fluorescence in the facial motor nuclei indicating a loss of respiratory-related rhythmic motor output. Absence of spontaneous rhythmic activity in the pBc at E16.5. Unilateral electrical and chemical stimulation of the pBc fails to induce bilateral bursts and instead produces only ispilateral responses in the vicinity of stimulation. Absence of fast synaptic events in the pBc area suggesting a lack of AMPA/kainate receptor-mediated synaptic processing Absence of fast synaptic events in the pBc area suggesting a lack of AMPA/kainate receptor-mediated synaptic processing.	Homz.	pBc	2	NO

Continued on next page

Table S5 continued from previous page

Gene	Phenotype	Genotype ID	Sex	Finding	Zigosity	Expressed in pBc or BS	Refs.	Model of human disease

BS: brainstem; USP.: unspecified; M.: male; F.: female; Homz.: homozygous; Con.: conditional; Het.: heterozygous; Com.: complex; pBc: pre-Bötzinger complex.
